# In Situ Dark Adaptation Enhances the Efficiency of DNA Extraction from Mature Pin Oak (*Quercus palustris*) Leaves, Facilitating the Identification of Partial Sequences of the 18S rRNA and Isoprene Synthase (*IspS*) Genes

**DOI:** 10.3390/plants6040052

**Published:** 2017-10-24

**Authors:** Csengele E. Barta, Bethany Bolander, Steven R. Bilby, Jeremy H. Brown, Reid N. Brown, Alexander M. Duryee, Danielle R. Edelman, Christina E. Gray, Chandler Gossett, Amie G. Haddock, Mackenzie M. Helsel, Alyssa D. Jones, Marissa E. Klingseis, Kalif Leslie, Edward W. Miles, Rachael A. Prawitz

**Affiliations:** Department of Biology, Missouri Western State University, 4525 Downs Drive, Agenstein-Remington Halls, St. Joseph, MO 64507, USA; bbolander@missouriwestern.edu (B.B.); sbilby@missouriwestern.edu (S.R.B.); jbrown71@missouriwestern.edu (J.H.B.); rbrown26@missouriwestern.edu (R.N.B.); aduryee@missouriwestern.edu (A.M.D.); dedelman@missouriwestern.edu (D.R.E.); cgray2@missouriwestern.edu (C.E.G.); cgossett@missouriwestern.edu (C.G.); ahaddock@missouriwestern.edu (A.G.H.); mhelsel1@missouriwestern.edu (M.M.H.); ajones51@missouriwestern.edu (A.D.J.); mklingseis@missouriwestern.edu (M.E.K.); kleslie@missouriwestern.edu (K.L.); emilesiii@missouriwestern.edu (E.W.M.); rprawitz1@missouriwestern.edu (R.A.P.)

**Keywords:** DNA extraction, pin oak (*Quercus palustris*), leaves, dark adaptation, secondary metabolites, PCR, gene identification, 18S rRNA, isoprene synthase (*IspS*)

## Abstract

Mature oak (*Quercus* spp.) leaves, although abundantly available during the plants’ developmental cycle, are rarely exploited as viable sources of genomic DNA. These leaves are rich in metabolites difficult to remove during standard DNA purification, interfering with downstream molecular genetics applications. The current work assessed whether in situ dark adaptation, to deplete sugar reserves and inhibit secondary metabolite synthesis could compensate for the difficulties encountered when isolating DNA from mature leaves rich in secondary metabolites. We optimized a rapid, commercial kit based method to extract genomic DNA from dark- and light-adapted leaves. We demonstrated that in situ dark adaptation increases the yield and quality of genomic DNA obtained from mature oak leaves, yielding templates of sufficiently high quality for direct downstream applications, such as PCR amplification and gene identification. The quality of templates isolated from dark-adapted pin oak leaves particularly improved the amplification of larger fragments in our experiments. From DNA extracts prepared with our optimized method, we identified for the first time partial segments of the genes encoding 18S rRNA and isoprene synthase (*IspS*) from pin oak (*Quercus palustris*), whose full genome has not yet been sequenced.

## 1. Introduction

Oaks (*Quercus* spp.) have been frequently reported to be some of the most challenging species to obtain high quality and quantity DNA from, as their leaves, shoots, and buds are particularly rich in carbohydrates, phenolic substances and secondary metabolites [1,2,3], contaminants that are difficult to remove during standard DNA isolation [4,5,6,7,8,9,10,11,12,13,14]. 

Healthy mature leaves, having achieved their full photosynthetic capacity, produce, and accumulate carbohydrates in high quantities during the photoperiod. These reserves are progressively depleted overnight via cellular respiration [15]. Light availability affects leaf phenolic content [16], as well as the rate of terpenoid synthesis, which is stimulated by light but ceases in the dark [17,18]. Mature oak leaves are rich in carbohydrates and their phenolic profile includes molecules such as gallotannins, ellagitannins, flavonals, catechol, resorcinol, hydroquinone, and pyrogallol [19,20]. North American oaks also synthesize terpenoid molecules, such as isoprene, while some clades of European oaks produce other terpenoids, for example, monoterpenes [21,22,23]. Therefore typically soft, non- photosynthesizing tissues (such as young roots, germinating seedlings, buds, flowers or fruits) or young, developing leaves are preferred for genomic DNA isolation from these species as they contain higher amounts of DNA and lower amounts of potential inhibitory metabolites than mature leaves [24,25,26,27]. However, these tissues are often available only briefly during the lifecycle of many long-lived woody species, unless they are maintained under controlled conditions or in vitro, in tissue, or protoplast cultures, which require time consuming and costly preparation, maintenance, and additional resources that often unavailable at education centered, applied learning institutions [28,29]. The season-long availability of mature leaves is often greater for many woody species of interest for genomic studies in temperate regions, or year-round in some species, such as live oaks (*Quercus virginiana*) in the Southern United States or the Japanese evergreen oaks (*Quercus acuta*) in Eastern Asia. However, their direct applications are limited due to the difficulties in isolating high quality and quantity DNA from the metabolite rich tissue [24,25,26,27]. 

Commercial kits are relatively ineffective in extracting DNA from mature leaves of many species of interest [10,11,12,13,14,24,25,26,27,30,31] and require time-consuming and costly pre- and/or post-processing of samples. This may include the digestion of cell walls, isolation of intact protoplasts, enzymatic break down of sugars, isolation of nuclei, mitochondria or chloroplasts, the concentration of the extract, or the separation of DNA from contaminants [3,5,32,33,34,35].

Oaks are particularly abundant in the state of Missouri, accounting for over 86% of the total hardwood forest coverage [36], and are the dominant woody species in North America, with 68% coverage consisting of 191 million acres [37]. Despite the high scientific interest in oak genomes, only the pedunculate oak’s (*Quercus robur*) genome has been sequenced to date, but these sequences have not been yet annotated, limiting gene discovery applications [38]. 

Difficulties in isolating high quantities of contaminant-free genomic DNA for downstream applications from leaves of species rich in sugars, polyphenolics, and terpenoids, prompt the urgent need to revisit, adapt and improve DNA isolation procedures. Ideally, procedures yielding high quality and quantity DNA would also eliminate the need of costly pre- and post-processing. The current study aimed to: (a) exploit day- and night-time metabolic differences in mature and young pin oak (*Quercus palustris*) leaves to adapt a rapid, commercial kit based DNA purification method to yield high quantity, contaminant-free genomic DNA suitable for downstream applications; (b) test whether the developed method can also improve the quality and quantity of DNA extracted from mature leaves of other species with metabolic properties similar to pin oak; and, (c) as proof of concept, use the DNA extracted from mature pin oak (*Q. palustris*) leaves to identify for the first time the partial sequences of the genes encoding the 18S rRNA (a DNA barcode-gene) and isoprene synthase (*IspS*) for future studies. Pin oak (*Q. palustris*) is native to and wide-spread in eastern and mid-western North America [37], but studies of this species have been limited by the lack of available genetic information.

We demonstrate that higher quality and quantity DNA can be obtained from in situ dark-adapted mature pin oak leaves, than from light-adapted, photosynthetically active healthy mature leaves. We show that templates obtained from dark-adapted leaves are suitable for downstream applications, such as fragment amplification or gene identification. We confirm that in situ dark adaptation also improves either the quantity, the quality, or (in some species) both the quantity and quality of DNA extracted from leaves of other species. Dark adaptation was especially beneficial to extractions from other *Quercus* species. 

The optimized method that we describe in this study is expected to provide a safe and easily applicable tool for genomic studies. The method is also appropriate for applied research and in-classroom experiences, due to the short time (less than one hour) required for completion. Rapid methods are often preferred in the applied learning laboratories of education-centered institutions employing solely undergraduate students. The method will also decrease sample processing costs, thus eliminating the need for pre- or post-processing and sample repeats, to gain sufficient contaminant-free DNA for downstream applications.

## 2. Results

### 2.1. DNA Extraction from Challenging, Contaminant-Rich Leaf Samples: In Situ Dark Adaptation Improves the Quality and Quantity of DNA Extractable from Mature Pin Oak (Q. palustris) Leaves

**Figure 1:**
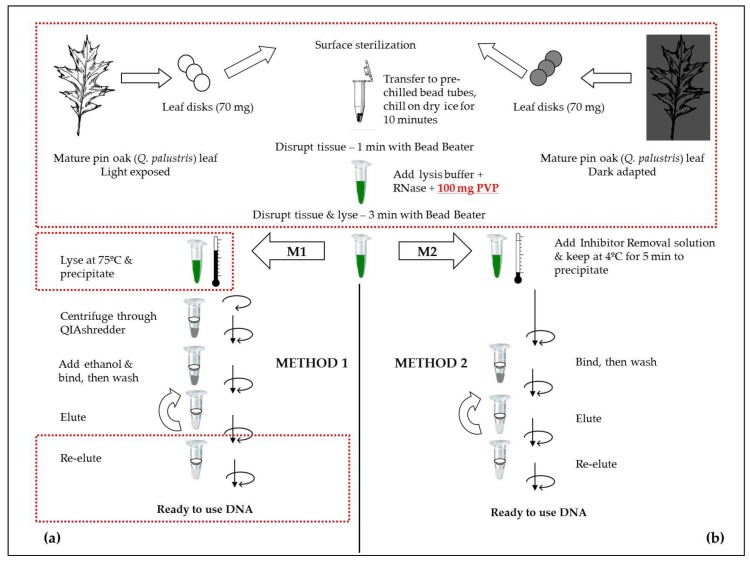
Schematic design of the methods adapted to extract genomic DNA from light or dark-adapted pin oak (*Q. palustris*) leaves or leaves of other species rich in polysaccharides and secondary metabolites: (**a**) M1, using CTAB (based on Qiagen DNeasy Plant DNA extraction kit) and (**b**) M2, using phenol (based on MoBio Power Plant DNA extraction kit) for contaminant removal. Red box framing indicates steps specifically modified within this work, differing from kit manufacturer recommendations.

We optimized two DNA extraction methods (M1 and M2, Figure 1) from commercial kits that are typically inappropriate for leaf samples of woody species rich in secondary metabolites and sugars (such as *Quercus palustris*) [24,25,26,39,40]. M1 is based on the DNeasy Plant DNA extraction kit (Qiagen, Hilden, Germany) using cetyl timethylammonium bromide (CTAB) for extraction, and M2 on the Power Plant extraction kit (MoBio, Carlsbad, CA, USA), precipitating contaminants with a phenol solution. The steps of the methods are described in Section 4 and schematically presented in Figure 1.

High quality DNA is characterized by DNA of high molecular weight, without contaminating substances, such as proteins, polysaccharides, phenolics, or other secondary metabolites [41]. The A_260/280nm_ absorbance ratio is typically used to assess the extent of protein contamination in DNA extracts (with protein absorbance peaking at 280 nm). An extract with an A_260/280nm_ ratio ≥ 1.8 is generally accepted as protein free. Significantly lower ratios may indicate the presence of proteins or other contaminants with strong absorbance at 280 nm [41,42,43,44]. A second measure of nucleic acid extract purity is given by the A_260/230nm_ absorbance ratio, with accepted values for this ratio ranging between 1.9 and 2.2 in pure nucleic acid extracts. Lower values indicate the presence of contaminants, such as carbohydrates or certain phenolics, which typically absorb at 230 nm [41,42,43,44].

**Figure 2:**
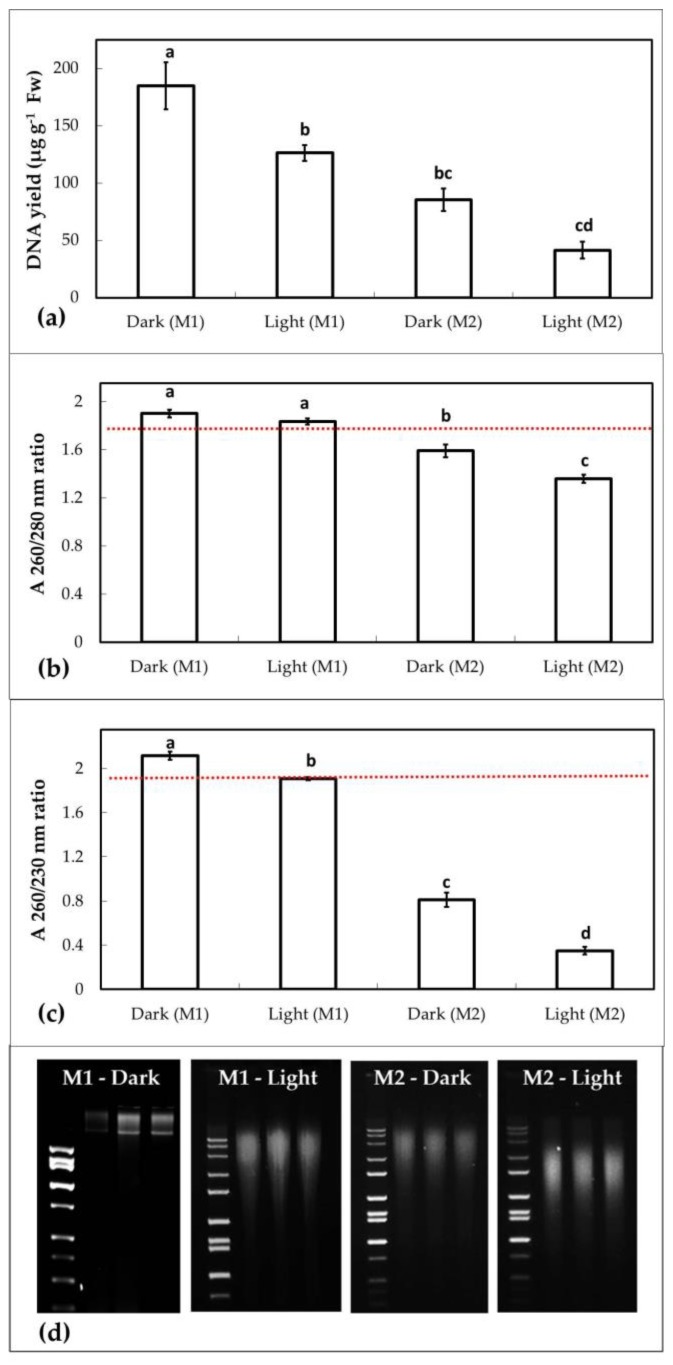
Quantity and quality of DNA extracted from mature pin oak (*Q. palustris*) leaves. (**a**) DNA yield, expressed as µg DNA g^−1^ fresh weight (Fw); (**b**) A_260/280nm_ ratios indicative of the presence or absence of protein-type contaminants in the DNA extract and (**c**) A_260/230nm_ ratios indicative of the presence or absence of carbohydrate or phenolic-type contaminants in the DNA extract were measured and/or calculated based on the absorbance at 230 nm, 260 nm, and 280 nm of DNA extracts prepared from in situ dark- or light-adapted leaves using the extraction methods M1 or M2. The red dotted line on panel (**b**) indicates the 1.8 and panel (**c**) the 1.9 threshold ratio for A_260/280nm_ and A_260/230nm_. Values above those thresholds indicate contaminant free DNA extracts. Data are shown as means ± standard error (SE) of five extractions (*n =* 5) each. Quantitative and qualitative differences between DNA extracted from dark- and light-adapted leaves with methods M1 and M2 were analyzed using one-way ANOVA with post-hoc Tukey HSD Test. Significant differences (at *p* < 0.01) are indicated by letters above bars. Gel images shown in panel (**d**) are indicators of the integrity of genomic DNA extracted using M1 and M2 on dark- and light-adapted mature leaves. The images show representative examples of independent extractions for each method (*n* = 3), obtained from dark- or light-adapted leaves. Each lane on the 2% agarose gel containing 0.5 µg/mL ethidium bromide contains 400 ng genomic DNA. DNA size ladders were of 2 kb and 10 kb.

We extracted the highest quantity (Figure 2a) and quality (Figure 2b,c) genomic DNA from mature, dark-adapted pin oak leaves using the modified protocol M1, in the presence of CTAB.

Using method M1, average DNA yield of dark-adapted leaves was of 184.8 ± 20.5 µg g^−1^ Fw, with A_260/280nm_ ratios of 1.9 ± 0.03 and A_260/230nm_ ratios of 2.11 ± 0.031 (SE, *n =* 5), indicating contaminant-free extracts. Light-adapted sample yields were significantly lower (*p* = 0.015), with 126.4 ± 6.8 µg DNA g^−1^ Fw (Figure 2a) The light-adapted extract was free of protein contaminants, as indicated by the A_260/280nm_ ratio of 1.83 ± 0.025 (Figure 2b), however, the A_260/230nm_ ratio of 1.9 ± 0.013 was significantly lower (*p* = 0.011) than that of dark-adapted leaves (Figure 2c). Yields of dark-adapted samples were higher than values reported by Qiagen for a representative species of the oak genus (European red oak, *Quercus robur*). For comparison, yields of light-adapted samples were within the 100–150 µg g^−1^ Fw range reported by Qiagen for young leaves (Qiagen, DNeasy procedure). Young leaves contain lower amounts of secondary metabolites, or enzymes capable of degrading DNA during purification, than mature leaves [45,46,47,48,49,50]. With method M2, the approach based on phenolic precipitation of contaminants, dark-adapted leaves yielded nearly 50% less genomic DNA (85.5 ± 9.7 µg g^−1^ Fw) than the samples extracted using M1 (Figure 2a). The dark-adapted A_260/280nm_ ratio of 1.59 ± 0.05 was significantly lower (*p* = 0.001) with method M2 than M1 (Figure 2b), indicating the presence of proteins or other contaminants absorbing at 280 nm, which may interfere with downstream applications. The dark-adapted A_260/230nm_ ratio of 1.81 ± 0.06 was also below the threshold for contaminant free extracts, indicating contamination with molecules absorbing at 230 nm (Figure 2c). Light-adapted samples extracted using method M2 yielded the lowest genomic DNA amount of all the samples (41.6 ± 7.3 µg g^−1^ Fw), with an A_260/280nm_ ratio of 1.35 ± 0.035 and A_260/230nm_ ratio of 0.348 ± 0.032, suggesting the presence of protein, carbohydrate, and/or phenolic contaminants in the extract (Figure 2). 

We also tested the integrity of the genomic DNA extracted from dark- and light-adapted leaves with both methods, using gel electrophoresis. DNA integrity was not compromised during the extraction when method M1 was used on dark-adapted samples. The genomic DNA in these samples appears as a large molecular mass band, with no indication of trailing to suggest sheering (Figure 2d). However, the integrity of DNA was compromised in light-adapted samples using method M1, as well as both dark- and light-adapted leaves using M2. The trailing on the gel indicates genomic DNA fragmentation, with large and smaller molecular mass fragments present in the samples (Figure 2d).

For comparison, we also extracted genomic DNA from young pin oak (*Q. palustris*) leaves, harvested early in the season, shortly after bud opening. With method M1, young leaves yielded 185.7 ± 32.8 µg g^−1^ Fw and 179.5 ± 21.4 µg g^−1^ Fw for dark- and light-adapted samples, respectively (*n =* 3). Yields from dark- and light-adapted young leaves were not significantly different (*p* = 0.6) and were comparable to DNA amounts isolated from mature, dark-adapted pin oak leaves using method M1. These results showed that dark adaptation particularly benefits mature leaves rather than young, developing leaves. Although method M2 was less efficient on young leaves than M1, surprisingly, the obtained yields were significantly higher (*p* = 0.001) than the yields obtained from mature leaves. Using M2, we obtained DNA yields of 142.8 ± 18.5 µg g^−1^ Fw and 104.2 ± 38.5 µg g^−1^ Fw from dark- and light-adapted samples. All of the samples were clean of contaminants (A_260/280nm_ ≥ 1.8 and A_260/230nm_ ≥ 1.8), with the exception of the genomic DNA extracted from light-adapted samples using method M2, with A_260/280nm_ ratios of 1.65 ± 0.017 and A_260/230nm_ ratios of 1.8 ± 0.029. 

Extractions with method M1 were also repeated from simultaneously harvested dark- and light-adapted mature leaves after three months storage at –80 °C. Freezing and storage up to three months did not affect the yield, purity, and integrity of extractable DNA, and extraction efficiency was similar to the extractions performed on fresh leaf material (192.8 ± 10 µg g^−1^ Fw and 157.5 ± 4.28 µg g^−1^ Fw from dark- and light-adapted samples).

We also extracted DNA from dark- and light-adapted mature leaves using the original protocols on which we based our methods M1 and M2, developed by Qiagen and MoBio, respectively. DNA yield from the original Qiagen protocol was significantly higher (*p* = 0.002) in dark-adapted mature leaves (52.7 ± 7 µg g^−1^ Fw) than in light-adapted mature leaves (26.4 ± 2.9 µg g^−1^ Fw), however, yields were nearly four times lower than the yields we obtained using M1 to extract DNA from mature leaves. In addition, A_260/280nm_ ≤ 1.6 and A_260/230nm_ ≤ 1.1 ratios indicated contamination. The procedure developed by MoBio was less efficient than the Qiagen method, with DNA yields between 20.5 ± 2.8 µg g^−1^ Fw and 17.8 ± 1.2 µg g^−1^ Fw from dark- and light-adapted samples (differences were not significant, *p* = 0.13). A_260/280nm_ and A_260/230nm_ ratios were ≤1.0, again suggesting sample contamination with proteins and other inhibitor metabolites. 

Finally, as our method M1 was based on modifications made to a kit developed from the traditional CTAB method, we also compared the quality and quantity of DNA extracted using our method M1 from mature leaves versus the traditional CTAB method [51,52]. DNA yield with the CTAB method was significantly (*p* = 0.002) higher from dark-adapted leaves (140.2 ± 17.4 µg g^−1^ Fw) than from light-adapted samples (100.3 ± 12.9 µg g^−1^ Fw), indicating that dark adaptation improves the efficiency of the CTAB method. Nevertheless, DNA yield from dark-adapted leaves was significantly (*p* = 0.001) higher with our method M1 (184.8 ± 20.5 µg g^−1^ Fw, Figure 2a) than with the CTAB method. A_260/280nm_ and A_260/230nm_ ratios using the CTAB method were comparable to values using M1 for dark- and light-adapted samples. 

### 2.2. Amplification of Chloroplast and Nuclear Gene Segments from Genomic DNA Extracted from Dark- and Light-adapted Pin Oak (Q. palustris) Leaves: Successful Amplification of Larger Fragments Requires DNA Templates of Higher Purity, than the Amplification of Smaller Fragments

**Figure 3:**
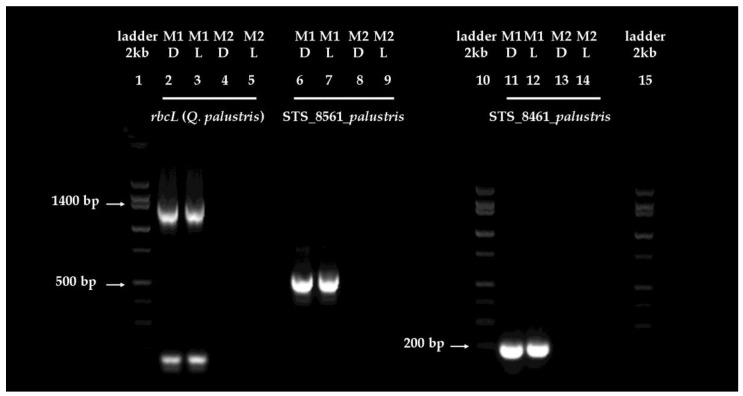
Amplification of selected target gene fragments from DNA extracted from in situ dark- or light-adapted mature pin oak (*Q. palustris*) leaves. The figure shows a representative example of PCR amplification of the 1300 bp *rbcL*, 500 bp STS_8561_*palustris* and 209 bp STS_8461_*palustris* sequence tagged site segments from dark- (D) and light-adapted (L) leaves, using extraction method M1 and M2, on a 2% agarose gel containing 0.5 µg/mL ethidium bromide. The low molecular weight bands (at about 175 bp) in the lane showing the amplified *rbcL* segment (M1D and M1L) are non-specific amplification products. A 2 kb DNA ladder was used for fragment sizing. Arrows indicate the position of the 1400, 500 and 200 bp bands in the marker line. To establish whether there is a quantitative difference between the efficiency of the amplification of fragments, apparent bands were excised from the gel and the DNA purified and quantified from the excised fragments. Yields of the 1300 bp *rbcL* amplicon fragments were of 325 and 227.6 ng, 500 bp STS_8561_*palustris* of 375 and 388 ng and 209 bp STS_8461_*palustris* of 420 and 426 ng, for dark- and light-adapted samples, respectively. No bands were observed for any of the samples using as template the DNA isolated by method M2, suggesting no amplification of the target gene fragments. Abbreviations stand for: D = dark-adapted; L = light-adapted; M1 = method 1; M2 = method 2.

Chloroplast- and nuclear-genomic target fragments were selected to test the suitability of genomic DNA extracts for fragment amplification in polymerase chain reactions (PCR). The three selected fragments were the larger, 1300 bp, chloroplast *rbcL* gene for ribulose 1,5-bisphosphate carboxylase/oxygenase large subunit (a DNA barcode gene); the medium size, 500 bp segment of the *Q. palustris* STS_(sequence tagged site) 8561; and the small 209 bp segment of *Q. palustris* STS_8461 segment. Testing DNA isolates from dark- and light-adapted mature pin oak (*Q. palustris*) leaves using methods M1 and M2 (Figure 1) suggested that the choice of methodology for genomic DNA extraction, and consequently, the quality of the obtained extracts (Figure 2b,c) affected fragment amplification (Figure 3). 

When genomic DNA extracted by method M1 was used as a PCR template, all three segments were successfully amplified (Figure 3). However, no amplification was observed when the template was the undiluted, protein, and carbohydrate contaminated genomic DNA stock (Figure 2b,c), obtained using method M2 for extraction (Figure 3). The lack of amplification in this case is indicative of the severe negative impact of impurities in the DNA extract on downstream applications, such as fragment amplification by PCR. 

**Figure 4:**
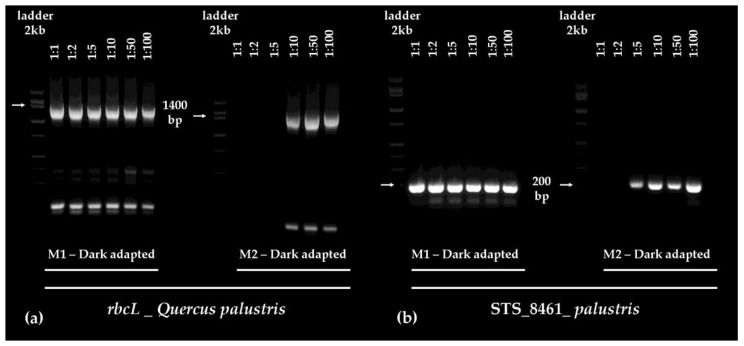
The impact of DNA template dilution on the amplification of the (**a**) 1300 bp *rbcL* and (**b**) 209 bp STS_8461_*palustris* fragments from DNA template extracted from in situ dark-adapted leaves using method M1 or M2. DNA templates were diluted in sterile, nuclease free water at ratios of 1:1, 1:2, 1:5, 1:10, 1:50, and 1:100, as denoted in the upper segment of the panels. 2 kb DNA ladder was used for amplicon size determination. Arrows point to the (**a**) 1400 bp and (**b**) 200 bp fragment position marker, for reference.

To quantify the amplicons of target fragments amplified from templates obtained from dark- and light-adapted leaves, all of the dominant band pairs (of 1300, 500, and 209 base pairs) were excised from the gels presented in Figure 3. DNA fragments were gel purified from the excised bands using a standard, highly reproducible procedure, developed by Macherey Nagel (NucleoSpin Gel- and PCR Clean Up kit), and the resulting DNA extracts were quantified. The amount of *rbcL* amplicon was higher in the templates from dark-adapted leaf extracts (325 ng DNA) than the templates from light-adapted leaf extracts (227.6 ng DNA). Yields of the STS_8561_*palustris* fragments (375 and 388 ng, dark- and light-adapted), or the STS_8461_*palustris* fragments (420 and 426 ng, dark- and light-adapted samples) were comparable. These results indicate that differences in the purity of DNA extracted from dark- or light-adapted mature leaves (Figure 2b,c) had a higher impact on the PCR amplification of larger, rather than smaller, fragments in our experiments. To assess whether the dilution of the lower quality template DNA extracted using method M2 from dark-adapted leaves (A_260/280nm_ of 1.59 ± 0.05 and A_260/230nm_ of 1.81 ± 0.06, Figure 2b,c), could relieve the inhibition of fragment amplification, the template was diluted in 1:1, 1:2, 1:5, 1:10, 1:50, and 1:100 ratios. For comparison, the high quality, contaminant free template DNA extracted from dark-adapted leaves using method M1 (A_260/280nm_ of 1.9 ± 0.03 and A_260/230nm_ of 2.11 ± 0.031, Figure 2b,c) was also diluted. Results indicate that the dilution did not affect the amplification of neither the larger 1300 bp *rbcL* fragment or the smaller 209 bp STS_8461_*palustris* fragment (Figure 4a,b) when the contaminant-free DNA stock extracted from dark-adapted leaves was used as concentrated or progressively diluted template. The dilution of the lower quality template allowed for better amplification, as expected, with the dilution reducing the amount of inhibitors in the sample. Interestingly however, the larger 1300 bp *rbcL* fragment required a more extensive dilution (1:10, Figure 4a) to amplify, than the small 209 bp STS_8461_*palustris* fragment (1:5, Figure 4b).

These results support our earlier finding (Figure 3, and DNA quantitative analysis) that higher quality DNA templates may be required for the PCR amplification of larger fragments (Figure 4). However, extracts with some contaminants can nonetheless be rescued and used in PCR applications, when DNA templates, and consequently impurities, are appropriately diluted depending on the molecular size of the expected fragment.

### 2.3. In Situ Dark Adaptation Improves the Quality and/or Quantity of DNA Extractable from Other Challenging, Contaminant Rich Mature Leaf Samples as Well

The impact of in situ dark adaptation on DNA yield and quality has previously been assessed in a variety of other plant species, with metabolic and biochemical profiles similar to that of pin oak (*Q. palustris*) [53,54,55,56,57,58,59,60,61]. The mature leaves of these species are also typically rich in polysaccharides, phenolics, terpenoids, and other secondary metabolites [48,49,56,57,58,59,60,61]. For extractions we used method M1 since it yielded the best results in extractions from mature pin oak leaves (Figure 2). We found that dark adaptation significantly improved the yield of DNA extracted from post oak (*Q. stellata*) (*p* = 0.001) and loblolly pine (*Pinus taeda*) (*p* = 0.002), while the differences were less accentuated in extracts prepared from the other tested species, American sweetgum (*Liquidambar styraciflua*), black poplar (*Populus nigra*), and black locust (*Robinia pseudoacacia*) (Table 1). For comparison, Qiagen reported significantly lower (*p* = 0.001) DNA yields of 200–250 µg g^−1^ Fw from young pine (*Pinus sylvestris*) leaves.

DNA extracted from dark-adapted samples of all tested species, except loblolly pine (*P. taeda*) was of higher quality (*p* < 0.01), than DNA deriving from light-adapted leaves. A_260/280nm_ was ≥1.8 and A_260/230nm_ ≥ 1.9 for all dark-adapted leaf isolates, while A_260/280nm_ ranged between 1.6 and 1.9 and A_260/230nm_ ranged between 1.17 and 1.9 in light-adapted mature leaf sample extracts (Table 1).

### 2.4. The Isolation and Identification of Partial Sequences for the Pin Oak (Q. palustris) 18S rRNA and Isoprene Synthase (IspS) Gene: Applications in Gene Identification and Phylogenetic Analysis

To validate the suitability of DNA extracted from mature leaves for downstream gene discovery applications, we directly amplified segments of the 18S rRNA and isoprene synthase (*IspS*) genes from templates extracted from dark-adapted mature pin oak (*Q. palustris*) leaves with method M1.

The genome of pin oak (*Q. palustris*) has not been sequenced yet, so we used unspecific primers for the amplification of both fragments. Primers were designed for known genomic segments for the same genes, of related species, relying on the assumption of a high degree of conservation and homology between genes and their RNA/protein products within plant clades. 

We successfully amplified for the first time a 930 bp segment of the gene 18S rDNA encoding for the 18S rRNA in pin oak (*Q. palustris*). The primers used for amplification were directed to the region spanning from (5′) 230 to 1150 bp (3′) positions of the northern red oak (*Quercus rubra*) gene (Accession # AF132892.1). Due to the high degree of homology and conservation of the gene encoding for the 18sRNA between oak species (near 99% sequence identity, BLAST), the selected primers amplified solely one fragment from the pin oak (*Q. palustris*) extract (Figure S1, inset I). The sequence of the amplified fragment was analyzed and identified as a segment of the 18S rRNA gene by sequencing and Basic Local Alignment Search Tool (BLAST) analysis.

A rooted phylogenetic tree was generated using available 18S rRNA gene sequences from species belonging to the Fabidae order, within the Rosids clade. Figure S2 shows the sequence alignment of the identified and database deposited (Accession # MF360746) pin oak (*Q. palustris*) 18S rRNA gene fragment, aligned with the corresponding regions of the 18S rRNA genes of other species selected for phylogeny analysis. The phylogram was generated from the aligned sequences using a maximum likelihood analysis algorithm. Based on the derived phylogeny, we found that the pin oak (*Q. palustris*) 18S rRNA gene shows the highest homology with, and is most closely related to the 18S rRNA genes of species from genera belonging to the Fagaceae family (Figures S1 and S2). 

We also successfully amplified and identified for the first time a 165 bp segment of the pin oak (*Q. palustris*) isoprene synthase (*IspS*) gene. The isoprene synthase gene (*IspS*) encodes for the terminal enzyme responsible for the production of isoprene (2-methyl-1,3-butadiene) in a variety of plant species. As no isoprene synthase genes have been sequenced to date from any of the species belonging to the oak (*Quercus*) genus we chose to use less specific primers, designed for a phylogenetically more distant relative of the pin oak (*Q. palustris*), the Australian pine tree (she-oak, *Casuarina equisetifolia*, Accession # LC006089.1) within the same monophyletic Rosids clade as the oaks [62]. The primers used for amplification were directed for the 165 bp segment spanning from the (5′) 935 to 1100 bp (3′) position of the Australian pine tree (*Casuarina equisetifolia*). 

**Figure 5:**
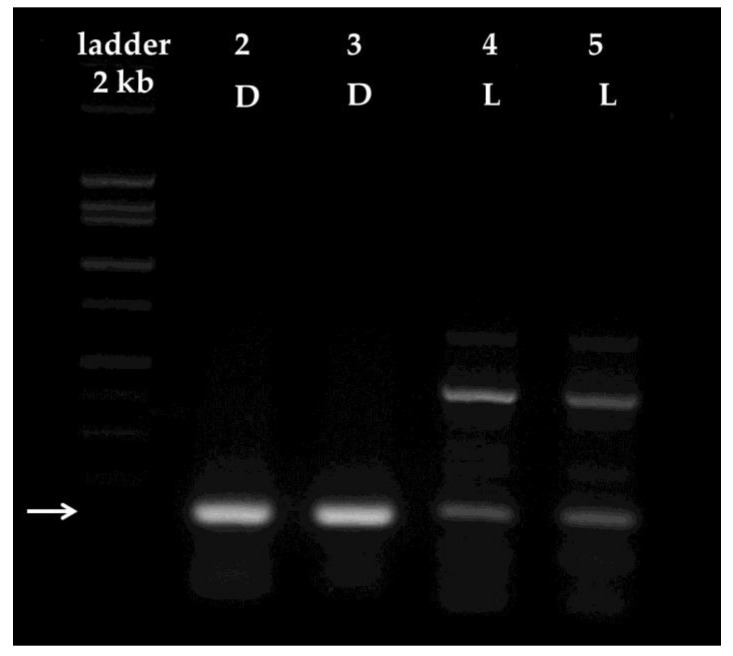
Amplification of a partial segment of the putative gene encoding for the isoprene synthase (IspS) enzyme. PCR products were separated on a 2% agarose gel stained with 0.5 µg/mL ethidium bromide. The arrow indicates the position of the 165 bp fragment of the putative *IspS* gene. Lanes 2 and 3 were loaded with representative reactions containing DNA templates isolated from dark-adapted leaves, using M1. Lanes 4 and 5 were loaded with representative reactions containing DNA templates isolated from light-adapted leaves, using M1. The white arrow annotates the position of the 165 bp fragments. D = dark-adapted; L = light-adapted.

The amplification resulted in a single fragment when M1 extracts from dark-adapted leaves were used as template (Figure 5). 

The amplified DNA fragment was gel-purified. We obtained 500 and 475 ng DNA from samples amplified from dark-adapted leaf template DNA (Figure 5, lanes 2 and 3), which was sufficient to meet sample submission standards for sequencing. When templates from light-adapted leaves were used, we obtained several fragments indicating unspecific amplification. Although we detected the weak amplification of a 165 bp fragment in samples deriving from light-adapted leaves, the amplicon that we could excise and purify from the gel was insufficient for sequencing reactions. We were only able to extract 95 and 75 ng DNA from the bands corresponding to the amplicons obtained from light-adapted samples (Figure 5, lanes 4 and 5), which was insufficient for sequencing. In addition, amplification was unsuccessful from any sample extracted using M2. 

**Figure 6:**
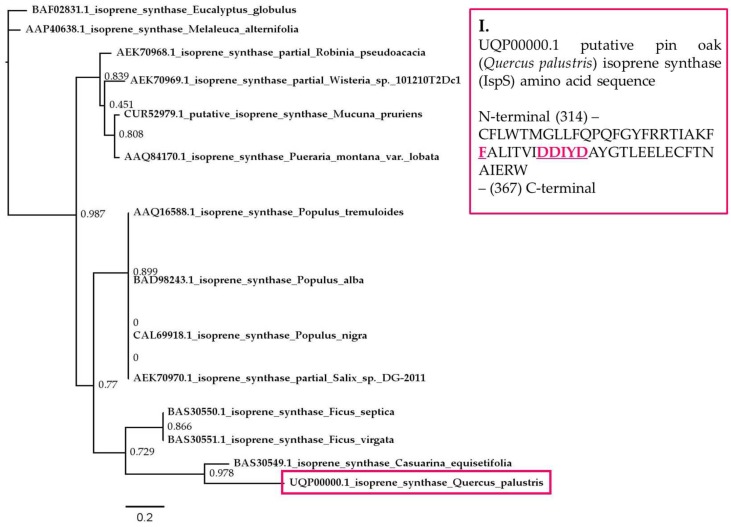
Phylogenetic relationship between the putative isoprene synthase (IspS) protein segment from pin oak (*Q. palustris*) identified in this study and other angiosperm isoprene synthase proteins. The phylogram was constructed using maximum likelihood analysis. Bootstrap analysis was done with 1000 replicates. The red colored box encloses the putative pin oak isoprene synthase (UQP00000.1 is an arbitrary annotation for the identified segment. Due to the size limitations imposed by GenBank for sequence submissions, the short *IspS* gene segment of 165 bp has not been deposited in the database). Numbers at nodes represent confidence values for splits. Branch length is proportional to the number of substitutions per site. The figure inset (**I**) shows the 53 amino acid sequence segment of the pin oak isoprene synthase (IspS) protein, identified in this study, spanning from the amino terminal (N-terminal) position 314 to the carboxy-terminal (C-terminal) position 367. The unique F338 amino acid and the conserved DDXXD motif are highlighted.

The identified *IspS* DNA sequence is provided in the Figure S3a. The sequence information was re-confirmed after designing specific primers based on the original sequence information, by re-amplifying and re-sequencing the segment. BLAST analysis indicated an 80% sequence similarity of our fragment with the corresponding isoprene synthase (*IspS*) segment of the Australian pine tree (*Casuarina equisetifolia*)*.* The gene segment was translated to the corresponding putative amino acid sequence of the isoprene synthase protein from pin oak (*Q. palustris*) (Figure 6, Inset I), and it was compared to and aligned with putative amino acid sequences of available isoprene synthase (IspS) proteins (Figure S3b). 

Sequence alignment confirmed that the selected 165 bp DNA encoded for a 53 amino acid long protein segment, spanning from positions 314 to 367 of the expected full length isoprene synthase enzyme. The segment is part of the putative catalytic site of the isoprene synthase enzyme [63]. In the position corresponding to the putative 338th amino acid for the full sequence, in our protein sequence is a phenylalanine (F338), an amino acid unique to isoprene synthase enzymes in the 338^th^ position (substituted most commonly by Isoleucine in other proteins) [63]. This confirms the identity of the segment as a putative isoprene synthase protein (Figure S3b and Figure 6, Inset I). The protein also contains the conserved DDXXD motif (in the case of the pin oak (*Q. palustris*) DDIYD) from position 345th to 349th in the amino acid sequence (Figure S3b and Figure 6, Inset I).

A phylogenetic analysis was performed using the maximum likelihood analysis algorithm. The phylogram showed that isoprene synthase proteins are diverse, with the putative pin oak (*Q. palustris*) isoprene synthase fragment showing the highest homology with the Australian pine tree (*Casuarina equisetifolia*) isoprene synthase. Surprisingly, the pin oak (*Q. palustris*) IspS showed a relatively low homology with isoprene synthases deriving from other woody species, such as poplar (*Populus* spp.) or willow (*Salix* spp.) (Figure 6).

## 3. Discussion

Developing methodologies, which facilitate the study of oak genomes is highly relevant to the scientific community and future studies. Therefore the goal of the current study was to optimize rapid commercial kit based procedures to overcome DNA isolation difficulties attributed to the physical and chemical properties of metabolite rich mature leaves and eliminate the need for costly pre- and/or post-processing steps. 

We optimized and tested two DNA purification methods (M1 and M2, Figure 1) suitable for DNA extraction from mature pin oak (*Q. palustris*) leaves. M1 was based on the DNeasy Plant DNA extraction kit (Qiagen) using CTAB for extraction and M2 on the Power Plant extraction kit (MoBio), which precipitates contaminants with a phenol solution. 

As the concentration of the inhibitors synthesized in mature oak leaves shows circadian variation [16,17,18,19,64,65,66,67], we hypothesized that in situ dark adaptation of mature leaves prior to DNA extraction could improve the yield and quality of DNA, in comparison with extracts prepared from light-adapted leaves. Our results demonstrate that dark adaptation improved the quantity and quality of DNA extracts using either method, but M1 was more efficient in extracting high quantity and quality DNA, in particular from dark-adapted mature leaves, than M2 (Figure 2). DNA integrity was preserved only in extracts prepared from dark-adapted leaves using M1 (Figure 2d). In addition, the DNA quantity isolated from dark-adapted leaves using M1 was significantly higher than the quantities extracted using the original kit methods and the traditional CTAB method. We also found that dark adaptation did not have a significant impact on the yield and quality of DNA prepared from young, developing leaves using M1. Leaf metabolic profiles change during their development, with young leaves accumulating lower carbohydrate amounts than mature leaves, and maximum secondary metabolite synthesis rates are reached only after full leaf expansion and maturation, which explains the differences we observed between young and mature leaf DNA extracts [45,46,47,48,49,50]. Of all the tested methods, M1 was the most efficient and dark adaptation benefitted in particular genomic DNA extractions from mature pin oak (*Q. palustris*) leaves using M1. DNA yields of the extracts prepared from light-adapted leaves using M1 and the traditional CTAB method were comparable, but dark adaptation significantly increased the yield of DNA in extracts prepared with the CTAB method as well. However, the original Qiagen procedure was relatively ineffective in mature oak leaf extractions. We also noted that M1 processing times were shorter (less than 1 h) than the time required for completing the traditional CTAB extraction (over 2.5 h). Short processing times make M1 a desirable alternative to the traditional CTAB method for classroom based, applied learning research projects, when students only have short laboratory periods available to perform the DNA isolation.

To test the suitability of DNA extracted from light- and dark-adapted mature pin oak leaves for PCR reactions, we amplified three gene segments (of 1300, 500, and 209 bp) from the chloroplast and nuclear genome (Figure 3). We confirmed that the extracts from dark- and light-adapted leaves prepared using M1 were of sufficient quality to allow for direct fragment amplification. However, differences in template quality affected in particular the amplification of the larger fragment (Figure 3). These data support earlier findings indicating that the quality of DNA template may be more critical when larger fragments are amplified [68]. We hypothesize that minimal contaminant carryover does not fully inhibit *Taq* polymerase activity, but may decrease the efficiency of PCR reactions amplifying larger fragments. We also do not exclude the potential effect of DNA fragmentation in extracts isolated from light-adapted mature leaves by M1 (Figure 2d), on PCR efficiency in our experiments. However, we exclude the possibility of an influence of gene copy numbers on the observed amplification differences of the compared fragments, in this case, as many copies of the chloroplast *rbcL* are present in plant cells and the chloroplast genome [69,70,71]. 

DNA templates isolated from mature pin oak leaves using M2 were of significantly lower quality than isolates obtained by M1 (Figure 2). The contaminants in these extracts fully inhibited the activity of the DNA polymerase, impeding the amplification of any of the three fragments in our experiments (Figure 3). In general, diluting contaminated DNA extracts to simultaneously decrease the concentration of inhibitors could “rescue” and restore PCR efficiency, but we found that success in amplifying larger fragments from our M2 isolates prepared from dark-adapted oak leaves may require more extensive dilutions than the amplification of smaller fragments. The amplification of the larger, 1300 bp chloroplast *rbcL* gene fragment required at minimum a 1:10 dilution of the template, while the smaller, 209 bp fragment was successfully amplified from a 1:5 dilution of DNA extracts (Figure 4). On the other hand, dilution did not have an effect on the amplification of *rbcL* and STS_8461_*palustris* fragments from DNA templates isolated using M1 from dark-adapted mature oak leaves (Figure 4). Diluting DNA extracts therefore may provide a suitable alternative for using less pure isolates in downstream applications if DNA concentrations are sufficiently high to allow for substantial dilution. Otherwise, post-processing, which may include DNA precipitation, may be required to concentrate the sample and remove contaminants. These data also support the critical role of template quality in sensitive PCR reactions, in particular, when the target segments are larger or present in low copy numbers.

Our data demonstrate that genomic DNA extraction from dark-adapted mature oak leaves using M1 developed in this study is a viable alternative to extractions from young leaves when young tissue is not available. While method M2 proved to be less efficient, a suitable amount of DNA can still be extracted using this method, in particular from dark-adapted mature leaves (Figure 2). Though the quality of M2 DNA extracts does not fit the most stringent quality requirements for downstream applications, appropriate dilution of the contaminated stock may be the sole post-processing step required to obtain DNA template suitable for PCR applications (Figure 2 and Figure 3). 

Testing the efficiency of M1 in other species with metabolic profiles similar to the pin oak [53,54,55,56,57,58,59,60,61,65,66,67,72,73] we found that dark adaptation improved the quality and/or quantity of mature leaf DNA extracts (Table 1). The applications of our method were most successful in species closely related to the pin oak (such as the post oak), but may require further species-specific refining of the extraction conditions for broader applications in other species.

In the current study we also successfully amplified and sequenced a 930 bp segment of the gene encoding for the 18S rRNA in pin oak (*Q. palustris*) (Accession # MF360746), for the first time (Figures S1 and S2). 

The 18S rDNA, the nuclear gene encoding for the 18S rRNA is one of the most frequently used genes in phylogenetic studies [69,74]. In conjunction with other chloroplast [69,70,71] and mitochondrial genes [75,76], the gene encoding for the 18S rRNA from all of the major angiosperm lineages played a particularly important role in establishing the main framework for angiosperm phylogeny, and served as a marker for biodiversity screening as a barcode gene. The 18S rRNA gene has also been described as an ideal internal control for reverse transcription polymerase chain reactions (RT-PCR), as its expression levels are constant [77]. We found that the pin oak (*Q. palustris*) 18S rRNA gene is highly homologous (with 99% sequence identity) with 18S rRNA genes of species from genera belonging to the Fagaceae family, forming a monophyletic group (Figure S1). 

The second gene of interest for our current work, the isoprene synthase gene (*IspS*), encodes for the terminal enzyme responsible for converting dimethylallyl pyrophosphate (DMAPP), and its isomer, isopentenyl pyrophosphate (IPP), to isoprene (2-methyl-1,3-butadiene, C_5_H_8_). Isoprene is a volatile biogenic hydrocarbon produced in the chloroplasts of a variety of plant species via the mevalonate-independent, 1-deoxy-d-xylulose 5-phosphate/methyl-erythritol 4-phosphate (DOXP/MEP) pathway [78]. Isoprene is released into the atmosphere in a light- and temperature-dependent manner [79,80], and is emitted from the leaves of many, but not all plant species. However, the capacity to synthesize isoprene has been shown to provide a variety of benefits to emitter species. In planta, isoprene acts to protect membranes from damages induced by high temperature stress that is commonly experienced by plants in their native growth environment on sunny days [50,81,82,83], and also protects plants from oxidative stress, potentially neutralizing short lived, damaging reactive oxygen species [84,85,86,87,88]. Isoprene emission from the terrestrial vegetation accounts for over 650 Tg of carbon that is released into the atmosphere annually [89,90]. In the troposphere, isoprene reactions may contribute to ozone formation, particularly in polluted environments [91], and may increase the lifetime of methane—a greenhouse gas—in the atmosphere with positive feedback on a warming climate [92]. 

In angiosperms, the capacity to emit isoprene has been hypothesized to have independently evolved multiple times [63], which would support the patchy distribution of the isoprene emission ability in various clades. From among oak species (*Quercus* spp.), all North American oaks emit isoprene, but many European oaks do not. Some clades of European oaks emit isoprene, while others emit other terpenoids as monoterpenes and a few species are non-emitters [21,22,23].

Sequences of the gene(s) encoding for isoprene synthase (IspS) and/or the isoprene synthase protein have been isolated from a variety of emitter species. These include poplars (*Populus alba*, *Populus* × *canescens*, *Populus tremuloides*, *Populus trichocarpa*, *Populus nigra*) [93,94,95,96], kudzu (*Pueraria montana*) [95], willow (*Salix glabra*) [Accession JQ943918.1], figs (*Ficus virgata*, *Ficus septica*), the Australian pine tree (*Casuarina equisetifolia*) [97], black locust (*Robinia pseudoacacia*), *Wisteria* sp. [63], and most recently *Arundo donax*, a herbaceous species [98]. Identified *IspS* sequences enable gene expression studies and the investigation of the evolution of isoprene synthesis and emission in plants. However, to date, no information is available on the isoprene synthase genomic or protein sequences of any oak species (*Quercus* spp.). 

In this work, we amplified, sequenced, and identified for the first time a 165 bp segment of the pin oak (*Q. palustris*) isoprene synthase (*IspS*) gene (Figure 5 and Figure S3a). We chose to sequence the gene using the gel purified fragments best amplified from DNA templates obtained from dark-adapted mature oak leaves using M1 developed in this study. As we used unspecific primers for the amplification, designed for a phylogenetically distant relative of oaks, we expected multiple unspecific fragments. Surprisingly, these reactions yielded a single band. We excised and purified the DNA fragment from the gel, which yielded sufficient fragment DNA to meet sequencing standards. On the other hand, we were unable to amplify in sufficient quantities for sequencing the 165 bp segment from light-adapted leaf templates extracted by M1. These reactions resulted in multiple, unspecific amplicons, as expected (Figure 5). Amplification was unsuccessful when templates isolated with M2 were used.

Isoprene synthase is part of the angiosperm (mono)terpene synthase-b (TpS-b) clade, hypothesized to have evolved from monoterpene synthases [63,98,99]. While isoprene synthase enzymes do not necessarily show high amino acid sequence similarity between species, all feature four unique amino acids (F338, S445, F484 and N505) in the catalytic region of the enzyme. These unique amino acids appear to function in sterically reducing the volume of the active site. Volume reductions diminish the possibility of an induced fit between the larger monoterpene precursor geranyl diphosphate (GDP) and the enzyme’s active site, selectively allowing only the binding of the smaller isoprene precursor to make isoprene, as opposed to other TpS-b enzymes, such as the monoterpene synthases [63,99]. Sequence alignment of the putative protein segment obtained by translating the sequenced pin oak (*Q. palustris*) genomic segment confirmed that the 53 amino acid long partial sequence spanning from the 314th to the 367th amino acid of the putative protein is a part of the enzyme’s catalytic site, with phenylalanine in position 338 (F338) (Figure S3b and Figure 6, Inset I). F338 is one of the four amino acids unique to isoprene synthase enzymes [63,99]. The protein also contains a highly conserved DDXXD motif downstream from F338 in positions spanning the 345th to 349th amino acids in the putative protein (Figure S3b and Figure 6, Inset I). 

The phylogram of IspS proteins revealed the highest homology between the putative pin oak (*Q. palustris*) isoprene synthase, and the corresponding segment of the Australian pine tree (*Casuarina equisetifolia*) isoprene synthase. Nevertheless, the *Quercus palustris* isoprene synthase (IspS) appears to be significantly different from other isoprene synthases deriving from woody species, such as *Populus* spp., *Salix* spp., or herbaceous taxa, to which kudzu (*Pueraria montana*) or velvet bean (*Mucuna pruriens*) belong (Figure S3b and Figure 6). Our initial observations, and the fact that minor changes in gene sequence can alter both the substrate and the product specificity of the isoprene synthase protein (IspS), are in support of prior hypotheses on the potential independent evolution of the isoprene emission trait in different clades as a result of convergent evolution driven by environmental constrains [63,95,99].

## 4. Materials and Methods

### 4.1. Plant Sample Collection, Surface Sterilization and/or Storage

Approximately 50-year-old pin oak (*Q. palustris*) trees were identified on the 700 acre green-space reserve of the Missouri Western State University Campus (St. Joseph, MO, USA, 39.7599° N, 94.7845° W). South-facing mature, fully developed leaves (4th or older) were selected and wrapped in aluminum foil in situ to darken the leaves overnight, from 5 PM until mid-day, the following day, while still attached to the branch. The foil was secured with paper clips, to prevent possible wind gusts from removing the foil cover. The darkening served to maximize the metabolic breakdown of accumulated sugar reserves. Adjacent leaves without foil cover were used as light-adapted controls, and both dark- and light-adapted leaves were harvested at mid-day. For comparison, young leaves freshly emerging from the buds were also collected, in the spring, from the same location and tree stand. Similarly, mature leaves of other species that are characterized by high sugar and polyphenolics concentrations were also harvested from the same site: post oak (*Quercus stellata*), American sweetgum (*Liquidambar styraciflua*), loblolly pine (*Pinus taeda*), black poplar (*Populus nigra*), and black locust (*Robinia pseudoacacia*).

For DNA extraction, three leaf disks of 1.5 cm^2^ (70 mg fresh weight) each per leaf were excised from the dark- and light-adapted leaves of the broad leaf species (*Q. palustris*, *Q. stellata*, *Liquidambar styraciflua*, *Populus nigra* and *Robinia pseudoacacia*) using a cork borer. From *Pinus taeda*, 70 mg fresh weight needle material was obtained. 

Leaf disks and needle segments were surface-sterilized to remove non-leaf related sources of DNA (such as bacterial or fungal DNA) and were washed in 95% ethanol for 5 s, 1% NaOCl for 2 min, followed by a wash in 70% ethanol for an additional 2 min [100]. Then the leaf material was rinsed multiple times using sterile water. Surface-sterilized samples were either processed fresh, or immediately frozen to –80 °C for later use, to test for storage shelf-life.

### 4.2. DNA Extraction and Quantification

Genomic DNA was extracted from in situ dark- and light-adapted leaves by modifying the initial key grinding, lysis, and few intermediate steps of the PowerPlant and DNeasy DNA isolation procedures developed by MoBio and Qiagen, respectively (Figure 1). We adapted the procedures for efficient, high quality, and quantity DNA extraction of rigid leaf samples that are rich in polysaccharides, phenolics, and other contaminants, conditions reported to lead to low yield in a variety of species with these kits [24,25,26,27,30]. We chose to modify and compare the procedures of two accessible commercial kits, the CTAB-based DNeasy Plant Mini Kit developed by Qiagen and the phenolic separation based MoBio Power Plant Kit developed by MoBio. 

The major modifications we made to the manufacturer-recommended protocols during method adaptation aimed to (a) maximize the in situ, pre-extraction removal of potential contaminating inhibitors of downstream applications, taking advantage of the natural differences in the metabolic status of dark- or light-adapted leaves; (b) enhance the break-down efficiency of the mechanically enforced, tough, mature leaf samples; and, (c) improve the efficiency of DNA collection and concentration during the final, DNA elution steps of the purification (Figure 1, box highlights). 

For extracting genomic DNA, standard procedures call for an initial mechanical grinding of the leaf sample in the presence of liquid nitrogen. Liquid nitrogen serves to aid the breakdown of the tissue as well as the accessing of the nuclear material without degradation. For the present application, we eliminated the need for liquid nitrogen and instead, prior to grinding, transferred the excised leaf disks into pre-chilled tubes containing metal beads, and maintained the samples on dry ice for an additional 10 min (Figure 1). 

All of the extraction tubes were pre-chilled on dry ice for 15 min prior to leaf material addition. Leaf disks and needle segments were pre-sliced to 1 mm^2^ slices using sterile razor blades and transferred to the pre-chilled tubes containing four metal beads of 2.38 mm (MoBio, Carlsbad, CA, USA) each, than kept on dry ice for 10 min. The frozen leaf material in the frozen tube was ground using a Mini-Beadbeater-24 (Biospec (BSP) Products Inc., Bartlesville, OK, USA) set to 3000 strokes/minute for 1 min. Lysis buffer and RNase were added to the pulverized leaf material, according to Qiagen or MoBio plant DNA extraction kit specifications. In addition, we supplemented both types of lysis buffers (provided by the manufacturer) with 100 mg PVP (polyvinyl pyrrolidone, MW 10,000, Sigma-Aldrich, St. Louis, MO, USA), to aid the removal of polyphenolics from the sample, and then ground the samples for an additional three minutes using the Mini Beadbeater-24. The homogenates were further processed using modified steps from the standard protocols developed for the DNeasy Plant Mini Kit (Qiagen, Hilden, Germany) and MoBio Power Plant Kit (MoBio, Carlsbad, CA, USA), as specified: 

Method # 1 (M1)—modified method based on the Qiagen DNeasy Plant Mini Kit: following sample disruption using the Mini-Beadbeater-24, samples were incubated at 75 °C for 10 min, instead of 65 °C as recommended by the manufacturer, mixing the sample thoroughly every 2 min using a vortex. In addition, samples were incubated for an additional 5 min, with an intense final mixing at the end of the 5 min (step 2, Quick Start protocol, Qiagen). 

Note: Pre-tests indicated an increase in lysis efficiency and DNA recovery with the lysis performed at higher temperatures, for a prolonged time. Increasing the incubation time further than the additional 5 min used in this protocol did not increase efficiency and DNA recovery. 

In addition, all of the centrifugation steps requiring 6000× *g* were performed at 10,000× *g*. DNA was eluted from the column using 100 µL AE buffer (provided by the manufacturer, Step 11, Quick Start protocol, Qiagen), then re-loaded onto the filter and incubated at room temperature for an additional 5 min prior to centrifugation (modified Step 12, Quick Start protocol, Qiagen) to recover the DNA. 

Method # 2 (M2)—modified method based on the MoBio Power Plant Kit: following sample disruption, steps were followed as recommended by the manufacturer. 

Extractions were also performed following the original Qiagen and MoBio protocols, without modifications, as well as using the traditional CTAB method [51,52] for comparison with our method M1. 

The obtained DNA stock was quantified spectrophotometrically with optical density (OD) values at 230, 260 and 280 nm (A_230nm_, A_260nm_, A_280nm_) recorded, using a NanoDrop One Microvolume Spectrophotometer (NanoDrop, Thermo Fisher Scientific Inc., Waltham, MA, USA). 

DNA purity was assessed by calculating the A_260/280nm_ and A_260/230nm_ absorbance ratios, indicators of protein and polysaccharide contaminants, respectively [41,44,101]. The integrity of the extracted genomic DNA was verified by agarose gel electrophoresis. Intact DNA was expected to be of high molecular mass and concentrated in a single band, with no evidence of trailing to indicate sheering. 

### 4.3. Pin Oak (Q. palustris) Genomic DNA Extracts as Templates for PCR Applications

The DNA extracted from dark- or light-adapted mature pin oak (*Q. palustris*) leaves using the two modified protocols M1 and M2, was tested for suitability for downstream applications by comparing the efficiency of PCR in amplifying chloroplast- and nuclear gene segments, using custom designed primer pairs. A 1300 bp segment of the *Q. palustris* chloroplast *rbcL* gene for ribulose 1,5-bisphosphate carboxylase/oxygenase large subunit, a DNA barcode gene (Accession # AB125023; primers used: sense (forward) 5′-ATGAGTCGTAGGGAGGGAC-3′; antisense (reverse) 5′-CAATTCAATTAAGAGAACGAGCGG-3′), a 500 bp segment *Q. palustris* STS 8561_*palustris*, sequence tagged site segment (Accession # HF563213; primers used: sense (forward) 5′-GCTCCCATATTTCTGGCAAGC-3′; antisense (reverse) 5′-GGTTTTCACTTAGCTTTATACATCCATAGC-3′) and 209 bp segment of *Q. palustris* STS 8461_*palustris*, sequence tagged site segment (Accession # HF563331.1; primers used: sense (forward) 5′-CAGATGAAGCAAAAGCTGTAGGTTC-3′; antisense (reverse) 5′-CCCAAAGC TTGAGCAAGC-3′) were selected for amplification. 

PCR reactions were performed in a 20 µL reaction volume (containing 150 ng DNA template/sample, 10 µL GoTaq Green 2× Master Mix, and 1 µL of sense and antisense primers, each of 50 pmol/µL stock). 

For testing the impact of template and possibly, contaminant dilution on the efficiency of PCR amplification of the 1300 bp *rbcL* and 209 bp STS_8461_*palustris* segments, the genomic DNA isolated from dark-adapted leaves using both M1 and M2 methods was diluted in 1:1, 1:2, 1:5, 1:10, 1:50, and 1:100 ratios.

PCR conditions were optimized to account for primer melting temperatures and maximum amplifiable segment length. Cycling was performed by using the following parameters: initial denaturation at 94 °C for 10 min, 45 cycles of 15 s denaturation at 94 °C, 60 s annealing at 52 °C, 120 s elongation at 74 °C, and a final extension step at 74 °C for 5 min. PCR products were separated on a 2% agarose gel, stained with 0.5 µg/mL ethidium bromide (Sigma-Aldrich, St. Louis, MO, USA). The amplified bands were imaged with a CareStream Gel Logic 2200 Pro Imaging system (CareStream Health Solutions, Rochester, NY, USA). All Purpose Lo (2k) and/or All Purpose Hi-Lo (10k) DNA ladders (Bionexus Inc., Oakland, CA, USA) were used as references for amplicon size estimation. 

To quantify the amplified fragment DNA, and characterize differences between samples, we excised the bands from the agarose gel(s), and purified the DNA using the NucleoSpin Gel and PCR Clean-Up kit (Macherey Nagel GmbH & Co., Duren, Germany) according to the manufacturers’ standardized protocol. The DNA was quantified using a NanoDrop One Microvolume Spectrophotometer (NanoDrop, Thermo Fisher Scientific Inc., Waltham, MA, USA).

### 4.4. Applications in Gene Identification: Isolation of Partial Sequences of Genes Encoding for the 18S rRNA and Isoprene Synthase (IspS) from Pin Oak (Q. palustris)

To validate the suitability of DNA extracted from mature pin oak (*Q. palustris*) leaves in gene discovery applications, we isolated and sequenced, for the first time, partial segments of the 18S rRNA and isoprene synthase (*IspS*) genes, using as template DNA from dark-adapted mature leaves.

A primer pair (Sense: 5′-CTCGCTGCTCTGCTGATT-3′; Antisense: 5′-ATGTCTGGACCT GGTAAGTTTC-3′) was designed to match the known sequence for the 930 bp segment spanning from the (5′) strand’s 230 to 1150 bp (3′) position of the 18S rRNA gene from the northern red oak (*Quercus rubra*) (Accession # AF132892.1), of the oak (*Quercus*) genus and used the primers to amplify the corresponding segment from the 18S rRNA gene of *Q. palustris*.

Similarly, primers (Sense: 5′-GGTGGAAGAACACTGGCC-3′; Antisense: 5′-CCCATCTCTCGATAGCATTTG-3′) were designed for the 165 bp segment, spanning from 5′ position 935 to 1100 bp of the known isoprene synthase (*IspS*) of the Australian pine tree (she-oak, *Casuarina equisetifolia*) (Accession # LC006089.1). As no isoprene synthase genes have been sequenced to date from any of the species belonging to the oak (*Quercus*) genus, we chose a more distant relative of our species of interest that is within the same monophyletic Rosids clade [62] as a reference. 

All of the PCR reactions were performed in 20 µL reaction volume (containing 150 ng DNA template/sample, 10 µL GoTaq Green 2× Master Mix, and 1 µL of sense and antisense primers, each of 50 pmol/µL stock) and 0.6 µL of 25 mM sterile MgCl_2_.

Cycling conditions to amplify the 930 bp segment of 18S rRNA gene were set up as follows: initial denaturation at 94 °C for 10 min, 40 cycles of 15 s denaturation at 94 °C, 60 s annealing at 52 °C, 120 s elongation at 74 °C, and a final extension step at 74 °C for 5 min. The isoprene synthase (*IspS*) gene segment was amplified under similar conditions, except the number of cycles was increased to 45 and the annealing temperature lowered to 49 °C, to account for the potential weak or partial pairing of the used primers, directed for the known gene of a more distant relative of our species of interest. We opted for the use of the above, less specific primer set instead of using degenerate primers due to the difficulties met in sequencing the amplicon using degenerate primers in performed pre-tests.

The amplified PCR products were separated on a 2% agarose gel and stained with 0.5 µg/mL ethidium bromide (Sigma-Aldrich, St. Louis, MO, USA) and the bands were imaged with a CareStream Gel Logic 2200 Pro Imaging system (CareStream Health Solutions, Rochester, NY, USA). All Purpose Lo (2k) and/or All Purpose Hi-Lo (10k) DNA ladders (Bionexus Inc., Oakland, CA, USA) were used as references for amplicon size estimation. 

The amplified fragments were excised from the agarose gel and purified using the NucleoSpin Gel and PCR Clean-Up kit (Macherey Nagel GmbH & Co., Duren, Germany) according to the manufacturer’s instructions. The gel-purified DNA was quantified using a NanoDrop One Microvolume Spectrophotometer (NanoDrop, Thermo Fisher Scientific Inc., Waltham, MA, USA). The exact sequence of the obtained candidate PCR fragments was determined by Sanger-sequencing using the services provided by Eurofin Genomics (Eurofin Genomics, LLC., Louisville, KY, USA, https://www.eurofinsgenomics.com/en/home.aspx), using the same primers used for their amplification. The samples were sequenced in multiplicates (*n =* 3) in both sense and antisense directions. 

In addition, after the initial sequencing of the 165 bp fragment of the gene encoding for the pin oak (*Q. palustris*) isoprene synthase, based on initial sequence information, an additional set of specific primers was designed (sense: 5′-GTGTTTCTTATGGACAATGGGATTA-3′; antisense: 5′-CTCGATAGCATTTGTGAACACTC-3′) and the fragment was re-amplified and re-sequenced to re-confirm the sequence information.

Basic Local Alignment Search Tool (BLAST) engine-powered searches of the GenBank Nucleotide Database of NCBI (https://blast.ncbi.nlm.nih.gov/Blast.cgi) were performed using the nucleotide sequences resolved through sequencing, confirming the identity of the isolated 18S rRNA gene segment and the 165 bp isoprene synthase (*IspS*) gene segment. The identified sequence for the 18S rRNA gene was deposited in the NCBI Gen Bank under the Accession # MF360746. Due to sequence size limitations for GenBank submissions (≥200 bp), the short isoprene synthase (*IspS*) sequence was not deposited in the database.

The putative isoprene synthase (*IspS*) gene segment was also translated to the encoded amino acid sequence using the ExPASy Tool (SIB, Swiss Institute of Bioinformatics), and screened for one of the four amino acids within the range of our identified sequence segment (F338), described to be unique to isoprene synthase proteins (IspS), differentiating them from related homologous terpene synthases (TpS) [63].

CLUSTAL*W* [102] (K-Align, http://www.ebi.ac.uk/Tools/msa/kalign/, European Bioinformatics Institute, EMBL-EBI) was used for multiple sequence alignment of the 18S rRNA DNA sequence and isoprene synthase protein sequence. The phylogenetic trees of the DNA sequence encoding for the 18S rRNA and isoprene synthase protein sequences were constructed using the phylogeny algorithm sequence, based on maximum likelihood analysis, developed and powered by the Laboratoire d’Informatique, de Robotique et Microelectronique de Montpellier, France (LIRMM, http://www.phylogeny.fr/) [103,104,105,106]. Bootstrap analysis was done with 1000 replicates. The phylogram was plotted using the FigTree 1.4.3 software (http://tree.bio.ed.ac.uk/software/Figuretree). 

### 4.5. Statistical Analysis

Quantitative and qualitative differences (total DNA yield, A_260/280nm_, and A_260/230nm_ yield ratio differences) between DNA extracted from dark- and light-adapted pin oak (*Q. palustris*) leaves with methods M1 and M2 were analyzed using one-way ANOVA with post-hoc Tukey HSD Test at a *p* < 0.01 significance level, with a sample size of five extractions each (*n =* 5). For all of the other species, a paired *t*-test was conducted at a *p* < 0.01 significance level on a sample size of *n =* 3. Data are shown as means ± standard error (SE).

## 5. Conclusions

In summary, we demonstrated that high quantity and quality genomic DNA can be extracted from abundantly available mature oak leaves (and leaves of a variety of other species), in particular, using the extraction method M1 we developed in this work. Though mature leaves were rarely used for DNA extractions in the past due to their high carbohydrate and secondary metabolite content, we demonstrated that in situ dark adaptation of leaves increased the yield and improved the quality of the obtained extracts, as compared to the efficiency of extraction from light-adapted leaves. We attribute these differences to the diurnal vs. nocturnal metabolic differences of the studied species. We propose in situ dark adaptation or pre-dawn harvesting of mature leaves as an ample resource for future DNA extractions when the availability of other tissues is limited. When we tested our extracts for suitability for downstream applications, we were able to amplify both nuclear and chloroplast genes, which also indicated that the obtained clean extracts can readily be used to study possibly all genes, regardless of their genomic origin (nuclear, mitochondrial, chloroplastic), minimizing the need for genome isolation and pre-processing.

To validate our extraction method and DNA extract suitability for downstream molecular genomic applications, we identified for the first time segments of the genes encoding for the 18S RNA and isoprene synthase from pin oak (*Q. palustris*), a species whose genome has not been sequenced and from which only a few genes have been isolated to date.

The identification of the partial sequence of pin oak (*Q. palustris*) isoprene synthase (*IspS*) lays the groundwork for studying changes in the expression of the gene under a variety of environmental conditions, to better understand how the current- and expected-future climate may affect the synthesis and release of isoprene into the atmosphere. Expression studies are also expected to apply the novel information provided by elucidating the partial sequence of the gene encoding for the *Q. palustris* 18S rRNA, a suitable internal reference gene for RT-PCR applications. 

In addition, adding genomic information from other species to the collection of known IspS sequences holds a promise for answering many of the remaining questions related to the single origin or convergent evolution of the isoprene emission trait in the plant kingdom. 

## Figures and Tables

**Table 1:** Comparison of quantities and quality of DNA extracted from in situ dark- or light-adapted mature post oak (*Q. stellata*), American sweetgum (*Liquidambar styraciflua*), loblolly pine (*Pinus taeda*), black poplar (*Populus nigra*) and black locust (*Robinia pseudoacacia*) leaves using the extraction method M1. DNA yield is expressed as µg g^−1^ fresh (leaf) weight (Fw). A_260/280nm_ ratios are indicative of the presence or absence of protein-type contaminants in the DNA extract and A_260/230nm_ ratios of the presence or absence of carbohydrate or phenolic contaminants. Data are shown as means ± standard error (SE) of 3 extractions (*n =* 3) per species. Quantitative and qualitative differences between DNA extracted from dark- and light-adapted leaves were analyzed using a paired *t*-test, at a significance level of *p* < 0.01. Significant differences between dark/light treatments are denoted by the (*) asterisk for each species.

Species		DNA Yield (µg g^-1^ Fw)	A_260/280nm_	A_260/230nm_
Post oak (*Quercus stellata*)	Dark	209.85 ± 26.14 (*)	1.89 ± 0.02 (*)	1.91 ± 0.015 (*)
	Light	112.14 ± 17.14	1.78 ± 0.015	1.42 ± 0.011
American sweetgum (*Liquidambar styraciflua*)	Dark	110.57 ± 14.57	1.80 ± 0.01 (*)	1.9 ± 0.018 (*)
	Light	90.28 ± 12.85	1.6 ± 0.015	1.17 ± 0.01
Loblolly pine (*Pinus taeda*)	Dark	354.42 ± 31.85 (*)	1.99 ± 0.02	2. 1 ± 0.024
	Light	226.28 ± 21.42	1.91 ± 0.03	1.9 ± 0.016
Black poplar (*Populus nigra*)	Dark	153.14 ± 22.71	1.86 ± 0.027 (*)	1.97 ± 0.013 (*)
	Light	121.42 ± 14	1.7 ± 0.025	1.7 ± 0.02
Black locust (*Robinia pseudoacacia*)	Dark	254.28 ± 22.28	2.0 ± 0.027 (*)	2.0 ± 0.02
	Light	210 ± 16.42	1.77 ± 0.02	1.87 ± 0.03

## Supplementary Materials

The following are available online at www.mdpi.com/2223-7747/6/4/52/s1, Figure S1: Phylogenetic relationship between the gene encoding for the 18S rRNA fragment (18S rDNA) identified in this study from pin oak (*Q. palustris*) leaves (Accession # MF360746) and other 18S rDNA sequences from families of the Fagales order, Figure S2: DNA sequence alignment of the 18S rDNA segments used for phylogenetic analyses (tree presented in Figure S1), Figure S3: *IspS* DNA sequence and protein sequence alignment.
