# Supplementary material for: In Situ Dark Adaptation Enhances the Efficiency of DNA Extraction from Mature Pin Oak (Quercus palustris) Leaves, Facilitating the Identification of Partial Sequences of the 18S rRNA and Isoprene Synthase (IspS) Genes
Source: Plants (Basel). 2017 Oct 24;6(4):52. doi: 10.3390/plants6040052 (PMC5750628; doi:10.3390/plants6040052)
Supplement: Supplementary file 1 [file plants-06-00052-s001.pdf]

Supplementary Materials: In Situ Dark Adaptation Enhances the Efficiency of DNA Extraction from Mature Pin Oak (*Quercus palustris*) Leaves, Facilitating the Identification of Partial Sequences of the 18S rRNA and Isoprene Synthase (*IspS*) Genes

Csengele E. Barta, Bethany Bolander, Steven R. Bilby, Jeremy H. Brown, Reid N. Brown, Alexander M. Duryee, Danielle R. Edelman, Christina E. Gray, Chandler Gossett, Amie G. Haddock, Mackenzie M. Helsel, Alyssa D. Jones, Marissa E. Klingseis, Kalif Leslie, Edward W. Miles and Rachael A. Prawitz

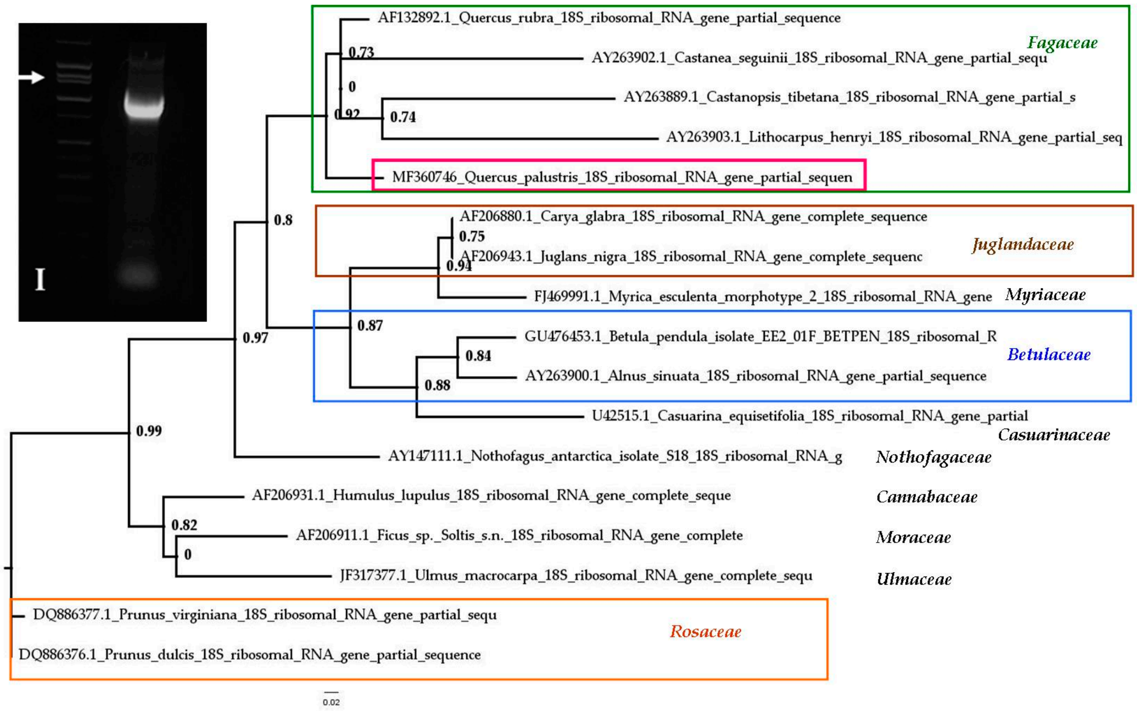

Figure S1. Phylogenetic relationship between the gene encoding for the 18S rRNA fragment (*18S rDNA*) identified in this study from pin oak (*Q. palustris*) leaves (Accession # MF360746) and other *18S rDNA* sequences from families of the Fagales order. The phylogenetic tree was constructed based on maximum likelihood analysis. Bootstrap analysis was done with 1,000 replicates. Colored boxes enclose sequences of representative species belonging to distinct families (labeled with color identical to that of the boxes). Numbers at nodes represent confidence values for splits. Branch length is proportional to the number of substitutions per site. The figure inset (**I**) shows a representative example of the amplified 930 bp fragment of the gene encoding for the 18S rRNA, in a 2% agarose gel stained with 0.5μg/mL ethidium bromide. The fragment was excised from the gel, the DNA purified and sequenced for genomic sequence identification. The white arrow annotates the position of the 1400 bp marker band.

```
AY263903.1      ------------------------------------------------------------
AY263889.1      ------------------------------------------------------------
AY263902.1      ------------------------------------------------------------
AF132892.1      ------TATGCTTGTCTCAAAGATTAAGCCATGCATGTGTAAGTATGAACTAATTCAGAC
MF360746_Q      ---TCATATGCTTGTCTCAAAGATTAAGCCATGCATGTGTAAGTATGAACTAATTCAGAC
AF206931.1      -----ATATGCTTGTCTCAAAGATTAAGCCATGCATGTGTAAGTATGAACTAATTCAGAC
AF206911.1      --------TGCTTGTCTCAAAGATTAAGCCATGCATGTGTAAGTATGAACTAATTCAGAC
JF317377.1      --------TGCTTGTCTCAAAGATTAAGCCATGCATGTGTAAGTATGAACTAATTCAGAC
DQ886376.1      ------------------------------------------------------------
DQ886377.1      ------------------------------------------------------------
AY147111.1      ------------------------------------------------------------
U42515.1_C      --------------------AGANTAAGCCATGCATGTGTAAGTATGAACTAATTCAGAC
AY263900.1      ------------------------------------------------------------
GU476453.1      ------------------------------------------------------------
AF206943.1      TAGTCATATGCTTGTCTCNAAGATTAAGCCATGCATGTGTAAGTATGAACTAATTCAGAC
FJ469991.1      --TGCAAGCGAGCGTGTCCGTATGTGAGCC-TGCATGTGTAAGTATGAACTAATTCAGAC
AF206880.1      ---TCATATGCTTGTCTCAAAGATTAAGCCATGCATGTGTAAGTATGAACTAATTCAGAC

AY263903.1      ------------------------------------------------------------
AY263889.1      ------------------------------------------------------------
AY263902.1      ------------------------------------------------------------
AF132892.1      TGTGAAACTGCGAATGGCTCATTAAATCAGTTATAGTTTGTTTGATGGTACCTGCTACTC
MF360746_Q      TGTGAAACTGCGAATGGCTCATTAAATCAGTTATAGTTTGTTTGATGGTATCTGCTACTC
AF206931.1      TGTGAAACTGCGAATGGCTCATTAAATCAGTTATAGTTTGTTTGATGGTATCTGCTACTC
AF206911.1      TGTGAAACTGCGAATGGCTCATTAAATCAGTTATAGTTTGTTTGATGGTACCTTCTACTC
JF317377.1      TGTGAAACTGCGAATGGCTCATTAAATCAGTTATAGTTTGTTTGATGGTATCTACTACTC
DQ886376.1      -----------------------TAATCAGTTATAGTTTGTTTGATGGTATCTACTACTC
DQ886377.1      -----------------------TAATCAGTTATAGCTTGTTTGATGGTATCTACTACTC
AY147111.1      ------------------------------------------------------------
U42515.1_C      TGTGAAACTGCGAATGGCTCATTAAATCAGTTATAGTTTGTTTGATGGTATCTGCTACTC
AY263900.1      ------------------------------------------------------------
GU476453.1      ------------------------------------------------------------
AF206943.1      TGTGAAACTGCGAATGGCTCATTAAATCAGTTATAGTTTGTTTGATGGTATCTGCTACTC
FJ469991.1      TGTGAAACTGCGAATGGCTCATTAAATCAGTTATAGTTTGTTTGATGGTATCTGCTACTC
AF206880.1      TGTGAAACTGCGAATGGCTCATTAAATCAGTTATAGTTTGTTTGATGGTATCTGCTACTC

AY263903.1      ------------------------------------------------------------
AY263889.1      --------------------------------------------------TGGAAGGGAT
AY263902.1      ------------------------------------------------------------
AF132892.1      GGATAACCGTAGTAATTCTAGAGCTAATACGTGCAACAAACCCCGACTTCTGGAAGGGAT
MF360746_Q      GGATAACCGTAGTAATTCTAGAGCTAATACGTGCAACAAACCCCGACTTCTGGAAGGGAC
AF206931.1      GGATAACCGTAGTAATTCTAGAGCTAATACGTGCAACAAACCCCGACTTCTGGAAGGGAT
```

```
AF206911.1    GGATAACCGTAGTAATTCTAGAGCTAATACGTGCAACGAACCCCGACTTCCGGAAGGGAT
JF317377.1    GGATAACCGTAGTAATTCTAGAGCTAATACGTGCAACAAACCCCGACTCATGGAAGGGGC
DQ886376.1    GGATAACCGTAGTAATTCTAGAGCTAATACGTGCAACAAACCCCGACTTCTGGAAGGGAT
DQ886377.1    GGATAACCGTAGTAATTCTAGAGCTAATACGTGCAACAAACCCCGACTTCTGGAAGGGAT
AY147111.1    -------------------------------------------------ACTGTAAGGGAT
U42515.1_C    GGATAACCGTAGTAATTCTAGAGCTAATACGTGCAACAAACCCCGACTTCTGGAAGGGAT
AY263900.1    ------------------------------------------------------------
GU476453.1    -----------------------------------------------------AAGGGAT
AF206943.1    GGATAACCGTAGTAATTCTAGAGCTAATACGTGCAACAAACCCCGACTTCTGGAAGGGAT
FJ469991.1    GGATAACCGTAGTAATTCTAGAGCTAATACGTGCAACAAACCCCGACTTCTGGAAGGGAT
AF206880.1    GGATAACCGTAGTAATTCTAGAGCTAATACGTGCAACAAACCCCGACTTCTGGAAGGGAT

AY263903.1    ------------------------------------------TCTGCTGATTCATGATAA
AY263889.1    GCATTTATTAGATAAAAGGCCGACGCGGGCTTTACGCTCTGCTCTGCTGATTCATGATCA
AY263902.1    -----------ATAAAAGGCCGACGCGGGCTCTGCTCGCTGCTCTGCTGATTCATGATAA
AF132892.1    GCATTTATTAGATAAAAGGCNGACGCGGGCTCTGCTCGCTGCTCTGCTGATTCATGATAA
MF360746_Q    GCATTTATTAGATAAAAGGTCGACGCGGGCTTAGCCCGTTGCTCTGATGATTCATGATAA
AF206931.1    GCATTTATTAGATAAAAGGTCGACGCGGGCTCTGCCCGTTGCTCTGATGATTCATGATAA
AF206911.1    GCATTTATTAGATAAAAGGTCGACGCGGGCTCTGCCCGTTGCCCTGATGATTCATGATAA
JF317377.1    GCATTTATTAGATAAAAGGTCGACGCGGGCTCTGCCCGTTGCTCTGATGATTCATGATAA
DQ886376.1    GCATTTATTAGATAAAAGGCTGACGCGGGCTCTGCCCGTTGCTCTGATGATTCATGATAA
DQ886377.1    GCATTTATTAGATAAAAGGCTGACGCGGGCTCTGCCCGTTGCTCTGATGATTCATGATAA
AY147111.1    GCATTTATTAGATAAAAGGTCGACGCGGGCTTTGCCCGTTGCTCTGATGATTCATGATAA
U42515.1_C    GCATTTATTAGATAAAAGGTCGACGCGGGCTCTGCCCGTTGCTCTGATGATTCATGATAA
AY263900.1    -TCTTTATTAGATAAAAGGTCGACGCGGGCTCTGCCCGTTGCTCTGATGATTCATGATAA
GU476453.1    GCATTTATTAGATAAAAGGTCGACGCGGGCTCTGCCCGTTGCTCTGATGATTCATGATAA
AF206943.1    GCATTTATTAGATAAAAGGTCGACGCGGGCTTTGCCCGTTGCTCTGATGATTCATGATAA
FJ469991.1    GCATTTATTAGGTAAAAGGTCGACGCGGGCTTTGCCCGTTGCTCTGATGATTCATGATAA
AF206880.1    GCATTTATTAGATAAAAGGTCGACGCGGGCTTTGCCCGTTGCTCTGATGATTCATGATAA
                                                         *** *********** *
AY263903.1    CTCGACGGATCGCACGGCCATCGTGCCGGCGACGCATTATTCAAATTTCTGCCCTATCAA
AY263889.1    CTCCACAGATCGCACGGACATCGTGCCGGAGACGCATTATTCAAATTTCTGCTCTATCAA
AY263902.1    CTCGACGGATCGCACGGCCATCGTGCCGGCGACGCATCATTCAAATTTTTGCCCTATCAA
AF132892.1    CTCGACGGATCGCACGGCCATCGTGCCGGCGACGCATCATTCAAATTTCTGCCCTATCAA
MF360746_Q    CTCGACGGATCGCACGGCCATCGTGCCGGCGACGCATCATTCAAATTTCTGCCCTATCAA
AF206931.1    CTCGACGGATCGCACGGCCTTCGTGCCGGCGACGCATCATTCAAATTTCTGCCCTATCAA
AF206911.1    CTCGACGGATCGCACGGCCACCGTGCCGGCGACGCATCATTCAAATTTCTGCCCTATCAA
JF317377.1    CTCGACGGATCGCAAGGCCATCGTGCCGGCGACGCATCATTCAAATTTCTGCCCTATCAA
DQ886376.1    CTCGACGGATCGCACAGCCATCGTGCTGGCGACGCATCATTCAAATATCTGCCCTATCAA
DQ886377.1    CTCGACGGATCGCACAGCCATCGTGCTGGCGACGCATCATTCAAATATCTGCCCTATCAA
AY147111.1    CTCGACGGATCGCACGGCCATCGTGCCGGCGACGCATCATTCAAATTTCTGCCCTATCAA
U42515.1_C    CTCGACGGATCGCACGGCCATCGTGCCGGCGACGCATCATTCAAATTTCTGCCCTATCAA
```

```
AY263900.1      CTCGACGGATCGCACGGCCATCGTGCCGGCGACGCATCATTCAAATTTCTGCCCTATCAA
GU476453.1      CTCGACGGATCGCACGGCCATCGTGCCGGCGACGCATCATTCAAATTTCTGCCCTATCAA
AF206943.1      CTCGACGGATCGCACGGCCATCGTGCCGGCGACGCATCATTCAAATTTCTGCCCTATCAA
FJ469991.1      CTCGACGGATCGCAAGGCCATCGTGCCGGCGACGCATCATTCAAATTTCTGCCCTATCAA
AF206880.1      CTCGACGGATCGCACGGCCATCGTGCCGGCGACGCATCATTCAAATTTCTGCCCTATCAA
                *** ** *******  * *  ***** ** ******* ******** * *** *******
AY263903.1      CTTTCAATGGAAGGATAATGG-CCTACTATGGTGGTGACGGGTGACGGAAAATTAGGGTT
AY263889.1      CTTTCTATGGTAAGATAGTGG-TCTACTATGGTGGTGACGGGTGACNGAAAATTAGGGTT
AY263902.1      CTTTCGATGGTAAGATAGTGG-CCTACTATGGTGGGGACGGGTGACAGAGAATTACGGTT
AF132892.1      CTTTCGATGGTAGGATAGTGGTCNTAWTATGGTGGTGACGGGTGACGGAGAAWTAGGKTT
MF360746_Q      CTTTCGATGGTAGGATAGTGG-CCTACTATGGTGGTGACGGGTGACGGAGAATTAGGGTT
AF206931.1      CTTTCGATGGTAGGATAGTGG-CCTACTATGGTGGTGACGGGTGACGGAGAATTAGGGTT
AF206911.1      CTTTCGATGGTAGGATAGTGG-CCTACTATGGTGGTGACGGGTGNCGGAGAATTAGGGTT
JF317377.1      CTTTCGATGGTAGGATAGTGG-CCTACTATGGTGGTGACGGGTGACGGAGAATTAGGGTT
DQ886376.1      CTTTCGATGGTAGGATAGTGG-CCTACCATGGTGGTGACGGGTGACGGAGAATTAGGGTT
DQ886377.1      CTTTCGATGGTAGGATAGTGG-CCTACCATGGTGGTGACGGGTGACGGAGAATTAGGGTT
AY147111.1      CTTTCGATGGTAGGATAGTGG-CCTACTATGGTGGTGACGGGTGACGGAGAATTAGGGTT
U42515.1_C      CTTTCGATGGTAGGATAGAGG-CCTACTATGGTGGTGACGGGTGACGGAGAATTAGGGTT
AY263900.1      CTTTCGATGGTAGGATAGAGG-CCTACTATGGTGGTGACGGGTGACGGAGAATTAGGGTT
GU476453.1      CTTTCGATGGTAGGATAGAGG-CCTACTATGGTGGTGACGGGTGACGGAGAATTAGGGTT
AF206943.1      CTTTCGATTGTAGGATAGAGG-CCTACAATGGTGGTGACGGGTAACGGAGAATTAGGGTT
FJ469991.1      CTTTCGATTGTAGGATAGAGG-CCTACAATGGTGGTGACGGGTGACGGAGAATTAGGGTT
AF206880.1      CTTTCGATTGTAGGATAGAGG-CCTACAATGGTGGTGACGGGTAACGGAGAATTAGGGTT
                ***** ** * * ****  **   **  ******* *******  * ** ** ** * **
AY263903.1      CGATTCCGGAAAGGGAGCCTGAAAAACGGCTACCACATCCAAGGAAGGCAGCAGGCGCGC
AY263889.1      CTATTCCGGAGAGGGAGCCTGAGAAACGGCTACCACATCCAGGGAAGGCAGCAGGCGCGC
AY263902.1      CGATTGCGGAGAGGGAGCCTGCAAAACGGCTACCACATCCAAGGAAGGCAGCAGGCGCTC
AF132892.1      CGATTCCGGAGAGGGAGCNTGAGAAACGGGTACCACATCCAAGGAAGGCAGCAGGCGCGC
MF360746_Q      CGATTCCGGAGAGGGAGCCTGAGAAACGGCTACCACATCCAAGGAAGGCAGCAGGCGCGC
AF206931.1      CGATTCCGGAGAGGGAGCCTGAGAAACGGCTACCACATCCAAGGAAGGCAGCAGGCGCGC
AF206911.1      CGATTCCGGAGAGGGAGCCTGAGAAACGGCTNCCACATCCAAGGAAGGCAGCAGGCGCGC
JF317377.1      CGATTCCGGAGAGGGAGCCTGAGAAACGGCTACCACATCCAAGGAAGGCAGCAGGCGCGC
DQ886376.1      CGATTCCGGAGAGGGAGCCTGAGAAACGGCTACCACATCCAAGGAAGGCAGCAGGCGCGC
DQ886377.1      CGATTCCGGAGAGGGAGCCTGAGAAACGGCTACCACATCCAAGGAAGGCAGCAGGCGCGC
AY147111.1      CGATTCCGGAGAGGGAGCCTGAGAAACGGCTACCACATCCAAGGAAGGCAGCAGGCGCGC
U42515.1_C      CGATTCCGGAGAGGGAGCCTGAGAAACGGCTACCACATCCAAGGAAGGCAGCAGGCGCGC
AY263900.1      CGATTCCGGAGAGGGAGCCTGAGAAACGGCTACCACATCCAAGGAAGGCAGCAGGCGCGC
GU476453.1      CGATTCCGGAGAGGGAGCCTGAGAAACGGCTACCACATCCAAGGAAGGCAGCAGGCGCGC
AF206943.1      CGATTCCGGAGAGGGAGCCTGAGAAACGGCTACCACATCCAAGGAAGGCAGCAGGCGCGC
FJ469991.1      CGATTCCGGAGAGGGAGCCTGAGAAACGGCTACCACATCCAAGGAAGGCAGCAGGCGCGC
AF206880.1      CGATTCCGGAGAGGGAGCCTGAGAAACGGCTACCACATCCAAGGAAGGCAGCAGGCGCGC
                * *** **** ******* **  ****** * ********* **************** *
```

```
AY263903.1      AAATTACCCAATCCTGACACGGGGAGGTATTGACAATAAATAACAATACCGGGCTCTCAC
AY263889.1      AAATTACCCAATCCTGACACGGGGAGGTAGTGACAATAAATAACAATACCGGGCTCTCAC
AY263902.1      AAATTACCCAATCCTGACACGGGGAGGTAGTGACAATAAATAACAATACCGGGCTCTCAC
AF132892.1      AAATTACCCAATCCTRAYACGGGGAGGTAGTGACAWTAAATAACAATACCGGGCTCTCAC
MF360746_Q      AAATTACCCAATCCTGACACGGGGAGGTAGTGACAATAAATAACAATACCGGGCTCTCAC
AF206931.1      AAATTACCCAATCCTGACACGGGGAGGTAGTGACAATAAATAACAATACCGGGCTCT-AC
AF206911.1      AAATTACCCAATCCTGACACGGGGAGGTAGTGACAATAAATAACAATACCGGGCTCT-AC
JF317377.1      AAATTACCCAATCCTGACACGGGGAGGTAGTGACAATAAATAACAATACCGGGCTCT-AC
DQ886376.1      AAATTACCCAATCCTGACACGGGGAGGTAGTGACAATAAATAACAATACCGGGCTCT-TA
DQ886377.1      AAATTACCCAATCCTGACACGGGGAGGTAGTGACAATAAATAACAATACCGGGCTCA-TA
AY147111.1      AAATTACCCAATCCTGACACGGGGAGGTAGTGACAATAAATAACAATACCGGGCTCA-TT
U42515.1_C      AAATTACCCAATCCTGACACGGGGAGGTAGTGACAATAAATAACAATACCGGGCTCTTAT
AY263900.1      AAATTACCCAATCCTGACACGGGGAGGTAGTGACAATAAATAACAATACCGGGCTCTTAT
GU476453.1      AAATTACCCAATCCTGACACGGGGAGGTAGTGACAATAAATAACAATACCGGGCTCTTTA
AF206943.1      AAATTACCCAATCCTGACACGGGGAGGTAGTGACAATAAATAACAATACCGGGCTCTTAC
FJ469991.1      AAATTACCCAATCCTGACACGGGGAGGTAGTGACAATAAATAACAATACCGGGCTCTTAC
AF206880.1      AAATTACCCAATCCTGACACGGGGAGGTAGTGACAATAAATAACAATACCGGGCTCTTAC
                *************** * *********** ***** ********************

AY263903.1      GAGTCTGGTAATTGGAATGAGTACAATCTAAATCCCTTAACGAGGATCCATTGGAGGGCA
AY263889.1      GAGTCTGGTAATTGGAATGAGTACAATCTAAATCCCTTAACGAGGATCCATTGGAGGGCA
AY263902.1      GAGTCTGGTAATTGGAATGAGTACAATCTAAATCCCTTAACGAGGATCCATTGGAGGGCA
AF132892.1      GAGTMTGGTAATTGGAATGAGTACAATCTAAATCCCTTAACGAGGATCCATTGGAGGGCA
MF360746_Q      GAGTCTGGTAATTGGAATGAGTACAATCTAAATCCCTTAACGAGGATCCATTGGAGGGCA
AF206931.1      GAGTCTGGTAATTGGAATGAGTACAATCTAAATCCCTTAACGAGGATCCATTGGAGGGCA
AF206911.1      GAGTCTGGTAATTGGAATGAGTACAATCTAAATCCCTTAACGAGGATCCATTGGAGGGCA
JF317377.1      GAGTCTGGTAATTGGAATGAGTACAATCTAAATCCCTTAACGAGGATCCATTGGAGGGCA
DQ886376.1      GAGTCTGGTAATTGGAATGAGTACAATCTAAATCCCTTAACGAGGATCCATTGGAGGGCA
DQ886377.1      GAGTCTGGTAATTGGAATGAGTACAATCTAAATCCCTTAACGAGGATCCATTGGAGGGCA
AY147111.1      GAGTCTGGTAATTGGAATGAGTACAATCTAAATCCCTTAACGAGGATCCATTGGAGGGCA
U42515.1_C      GAGTCTGGTAATTGGAATGAGTACAATCTAAATCCCTTAACGAGGATCCATTGGAGGGCA
AY263900.1      GAGTCTGGTAATTGGAATGAGTACAATTTAAATCCCTTAACGAGGATCCATTGGAGGGCA
GU476453.1      GAGTCTGGTAATTGGAATGAGTACAATCTAAATCCCTTAACGAGGATCCATTGGAGGGCA
AF206943.1      GAGTCTGGTAATTGGAATGAGTACAATCTAAATCCCTTAACGAGGATCCATTGGAGGGCA
FJ469991.1      GAGTCTGGTAATTGGAATGAGTACAATCTAAATCCCTTAACGAGGATCCATTGGAGGGCA
AF206880.1      GAGTCTGGTAATTGGAATGAGTACAATCTAAATCCCTTAACGAGGATCCATTGGAGGGCA
                **** ********************** ********************************

AY263903.1      AGTCTGGTGCCAGCAGCCGCGGTTATTCCAGCTCCAATAGCGTATATTTAAGTCGTTGCA
AY263889.1      AGTCTGGTGCCAGCAGCCGCGGTAATCCCAGCTCCAATAGCGTATATTTAAGTTGTTGCA
AY263902.1      AGTCTGGTGCCAGCAGCCGCGGTAATTCCAGCTCCAATAGCGTATATTTAAGTTGTTGCA
AF132892.1      AGTCTGGTGCCAGCAGCCGCGGTAATTCCAGCTCCAATAGCGTATATTTAAGTTGTTGCA
MF360746_Q      AGTCTGGTGCCAGCAGCCGCGGTAATTCCAGCTCCAATAGCGTATATTTAAGTTGTTGCA
AF206931.1      AGTCTGGTGCCAGCAGCCGCGGTAATTCCAGCTCCAATAGCGTATATTTAAGTTGTTGCA
```

```
AF206911.1      AGTCTGGTGCCAGCAGCCGCGGTAATTCCAGCTCCAATAGCGTATATTTAAGTTGTTGCA
JF317377.1      AGTCTGGTGCCAGCAGCCGCGGTAATTCCAGCTCCAATAGCGTATATTTAAGTTGTTGCA
DQ886376.1      AGTCTGGTGCCAGCAGCCGCGGTAATTCCAGCTCCAATAGCGTATATTTAAGTTGTTGCA
DQ886377.1      AGTCTGGTGCCAGCAGCCGCGGTAATTCCAGCTCCAATAGCGTATATTTAAGTTGTTGCA
AY147111.1      AGTCTGGTGCCAGCAGCCGCGGTAATTCCAGCTCCAATAGCGTATATTTAAGTTGTTGCA
U42515.1_C      AGTCTGGTGCCAGCAGCCGCGGTAATTCCAGCTCCAATAGCGTATATTTAAGTTGTTGCA
AY263900.1      AGTCTGGTGCCAGCAGCCGCGGTAATTCCAGCTCCAATAGCGTATATTTAAGTTGTTGCA
GU476453.1      AGTCTGGTGCCAGCAGCCGCGGTAATTCCAGCTCCAATAGCGTATATTTAAGTTGTTGCA
AF206943.1      AGTCTGGTGCCAGCAGCCGCGGTAATTCCAGCTCCAATAGCGTATATTTAAGTTGTTGCA
FJ469991.1      AGTCTGGTGCCAGCAGCCGCGGTAATTCCAGCTCCAATAGCGTATATTTAAGTTGTTGCA
AF206880.1      AGTCTGGTGCCAGCAGCCGCGGTAATTCCAGCTCCAATAGCGTATATTTAAGTTGTTGCA
                *********************** ** ************************** ******
AY263903.1      GTTAAAAAGCTCGTAGTTGAACCTTGGGTTGGGCAGAGCGGTCCGCCCCTGGTGTGCACC
AY263889.1      GTTAAAAAGCTCGTAGTTGAACCTTGGGTTGGGCAGAGCGGTCCGCCCCTGGTGTGCACC
AY263902.1      GTTAAAAAGCTCGTAGTTGAATCTTGGGTTGGGCAGAGCGGTCCGCCCCTGGTGTGCACC
AF132892.1      GTTAAAAAGYTCGTAGTTGAACCTTGGGTTGGGCAGAGCGGTCCGCCCCTGGTGTGCACC
MF360746_Q      GTTAAAAAGCTCGTAGTTGAACCTTGGGTTGGGCAGAGCGGTCCGCCCCTGGTGTGCACC
AF206931.1      GTTAAAAAGCTCGTAGTTGGACCTTGGGTTGGGTCGATCGGTCCGCCTCCGGTGTGCACC
AF206911.1      GTTAAAAAGCTCGTAGTTGGACCTTGGGTTGGGTCGATCGGTCCGCCTCCGGTGTGCACC
JF317377.1      GTTAAAAAGCTCGTAGTTGGACCTTGGGTTGGGTCGATCGGTCCGCCTATGGTGTGCACC
DQ886376.1      GTTAAAAAGCTCGTAGTTGGACCTTGGGTTGGGTCGACCGGTCCGCCTCTGGTGTGCACC
DQ886377.1      GTTAAAAAGCTCGTAGTTGGACCTTGGGTTGGGTCGACCGGTCCGCCTCTGGTGTGCACC
AY147111.1      GTTAAAAAGCTCGTAGTTGGACCTTGGGTTGGGCAGGTCGGTCCGCCCCTGGTGTGCACC
U42515.1_C      GTTAAAAAGCTCGTAGTTGGATCTTGGGTTGGGCGGATCGGTCCGCCCCTGGTGTGCACC
AY263900.1      GTTAAAAAGCTCGTAGTTGGATCTTGGGTTGGGCAGATCGGTCCGCCCCTGGTGTGCACC
GU476453.1      GTTAAAAAGCTCGTAGTTGGATCTTGGGTTGGGCAGATCGGTCCGCCCCTGGTGTGCACC
AF206943.1      GTTAAAAAGCTCGTAGTTGGATCTTGGGTTGGGCAGAGCGGTCCGCCCCTGGTGTGCACC
FJ469991.1      GTTAAAAAGCTCGTAGTTGGATCTTGGGTTGGGCAGAGCGGTCCGCCCCTGGTGTGCACC
AF206880.1      GTTAAAAAGCTCGTAGTTGGATCTTGGGTTGGGCAGAGCGGTCCGCCCCTGGTGTGCACC
                ********* ********* * ***********  *  *********   **********
AY263903.1      GGTCTGCTCGTCCCTTCTACCGGCGATGCGCTCCTGGCCTTAACTGGCCGGGTCGTGCCT
AY263889.1      GGTCTGCTCGTCCCTTCTACCGGCGATGCGCTCCTGGCCTTAACTGGCCGGGTCGTGCCT
AY263902.1      GGTCTGCTCGTCCCTTCTACCGGCGATGCGCTCCTGGCCTTAACTGGCCGGGTCGTGCCT
AF132892.1      GGTCTGCTCGTCCCTTCTACCGGCGATGCGCTCCTGGCCTTAACTGGCCGGGTCGTGCCT
MF360746_Q      GGTCTGCTCGTCCCTTCTACCGGCGATGCGCTCCTGGCCTTAACTGGCCGGGTCGTGCCT
AF206931.1      GGTCGGCTCGTCCCTTCTACCGGCGATGCGCTCCTGGCCTTAATTGGCCGGGTCGTGCCT
AF206911.1      GGTCGGCTCGTCCCTTCTACCGGCGATGCGCTCCTGGCCTTAACTGGCCGGGTCGTGCCT
JF317377.1      GGTCGTCTCGTCCCTTCTACCGGCGATACGCTCCTGGCCTTAATTGGCCGGGTCGTGCCT
DQ886376.1      GGTCGGCTCGTCCCTTCTACCGGCGATACGCTCCTGGCCTTAATTGGCCGGGTCGTGCCT
DQ886377.1      GGTCGGCTCGTCCCTTCTACCGGCGATACGCTCCTGGCCTTAATTGGCCGGGTCGTGCCT
AY147111.1      GGCCCGCTCGTCCCTTCTACCGGCGATGCGCTCCTGGCCTTAATTGGCCGGGTCGTGCCT
U42515.1_C      GGTCCGCTCGTCCCTTCTGCCGGCGATGCGCTCCTGGCCTTAACTGGCCGGGTCGTGCCT
```

```
AY263900.1      GGTCCGCTCGTCCCTTCTACCGGCGATACGCTCCTGGTCTTAATTGGCCGGGTCGTGCCT
GU476453.1      GGTCCGCTCGTCCCTTCTACCGGCGATACGCTCCTGGTCTTAATTGGCCGGGTCGTGCCT
AF206943.1      GGTCTGCTCGTCCCTTCTACCGGCGATGCGCTCCTGGCCTTAACTGGCCGGGTCGTGCCT
FJ469991.1      GATCTGCTCGTCCCTTCTACCGGCGATGCGCTCCTGGCCTTAACTGGCCGGGTCGTGCCT
AF206880.1      GGTCTGCTCGTCCCTTCTACCGGCGATGCGCTCCTGGCCTTAACTGGCCGGGTCGTGCCT
                *  *  ************ ******** ********* ***** ****************
AY263903.1      CCGGTGCTGTTACTTTGAAGAAATTAGAGTGCTCAAAGCAAGCCTACGCTCTGGATACAT
AY263889.1      CCGGTGCTGTTACTTTGAAGAAATTAGAGTGCTCAAAGCAAGCCTACGCTCTGGATACAT
AY263902.1      CCGGTGCTGTTACTTTGAAGAAATTAGAGTGCTCAAAGCAAGCCTACGCTCTGGATACAT
AF132892.1      CCGGTGCTGTTACTTTGAAGAAATTAGAGTGCTCAAAGCAAGCCTACGCTCTGGATACAT
MF360746_Q      CCGGTGCTGTTACTTTGAAGAAATTAGAGTGCTCAAAGCAAGCCTACGCTCTGGATACAT
AF206931.1      CCGGTGCTGTTACTTTGAAGAAATTAGAGTGCTCAAAGCAAGCCTACGCTCTGTATACAT
AF206911.1      TCGGTGCTGTTACTTTGAAGAAATTAGAGTGCTCAAAGCAAGCCTACGCTCTGTATACAT
JF317377.1      CCGGTGCTGTTACTTTGAAGAAATTAGAGTGCTCAAAGCAAGCCTACGCTCTGTATACAT
DQ886376.1      CCGGTGCTGTTACTTTGAAGAAATTAGAGTGCTCAAAGCAAGCCTACGCTCTGGATACAT
DQ886377.1      CCGGTGCTGTTACTTTGAAGAAATTAGAGTGCTCAAAGCAAGCCTACGCTCTGGATACAT
AY147111.1      CCGGTGCTGTTACTTTGAAGAAATTAGAGTGCTCAAAGCAAGCCTACGCTCTGGATACAT
U42515.1_C      CCGGCGCTGTTACTTTGAAGAAATTAGAGTGCTCAAAGCAAGCCTACGCTCTGGATACAT
AY263900.1      CCGGTGCTGTTACTTTGAAGAAATTAGAGTGCTCAAAGCAAGCCTACGCTCTGTATACAT
GU476453.1      CCGGTGCTGTTACTTTGAAGAAATTAGAGTGCTCAAAGCAAGCCTACGCTCTGGATACAT
AF206943.1      CCGGTGCTGTTACTTTGAAGAAATTAGAGTGCTCAAAGCAAGCCTACGCTCTGTATACAT
FJ469991.1      CCGGTGCTGTTACTTTGAAGAAATTAGAGTGCTCAAAGCAAGCCTACGCTCTGTATACAT
AF206880.1      CCGGTGCTGTTACTTTGAAGAAATTAGAGTGCTCAAAGCAAGCCTACGCTCTGTATACAT
                 *** ************************************************ ******
AY263903.1      TAGCATGGGATAACATCATAGGATTTCGGTCCTATTCTGTTGGCCTTCGGGATCGGAGTA
AY263889.1      TAGCATGGGATAACATCATAGGATTTCGGTCCTATTCTGTTGGCCTTCGGGATCGGAGTA
AY263902.1      TAGCATGGGATAACATCATAGGATTTCGGTCCTATTCTGTTGGCCTTCGGGATCGGAGTA
AF132892.1      TAGCATGGGATAACATCATAGGATTTCGGTCCTATTCTGTTGGCCTTCGGGATCGGAGTA
MF360746_Q      TAGCATGGGATAACATCATAGGATTTCGGTCCTATTAAGTTGGCCTTCGGGATCGGAGTA
AF206931.1      TAGCATGGGATAACATCATAGGATTTCGGTCCTATTCTGTTGGCCTTCGGGATCGGAGTA
AF206911.1      TAGCATGGGATAACATCATAGGATTTCGGTCCTATTCTGTTGGCCTTCGGGATCGGAGTA
JF317377.1      TAGCATGGGATAACATCATAGGATTCCGGTCCTATTATGTTGGCCTTCGGGATCGGAGTA
DQ886376.1      TAGCATGGGATAACATCATAGGATTTCGGTCCTATTCTGTTGGCCTTCGGGATCGGAGTA
DQ886377.1      TAGCATGGGATAACATCATAGGATTTCGGTCCTATTCTGTTGGCCTTCGGGATCGGAGTA
AY147111.1      TAGCATGGGATAACATCATAGGATTTCGGTCCTATTCTGTTGGCCTTCGGGATCGGAGTA
U42515.1_C      TAGCATGGGATAACATCATAGGATTTCGGTCCTATTGTGTTGGCCTTCGGGATCGGAGTA
AY263900.1      TAGCATGGGATAACATCATAGGATTTCGGTCCTATTCTGTTGGCCTTCGGGATCGGAGTA
GU476453.1      TAGCATGGGATAACATCATAGGATTTCGGTCCTATTGTGTTGGCCTTCGGGATCGGAGTA
AF206943.1      TAGCATGGGATAACATCATAGGATTTCGGTCCTATTGTGTTGGCCTTCGGGATCGGAGTA
FJ469991.1      TAGCATGGGATAACATCATAGGATTTCGGTCCTATTGTGTTGGCCTTCGGGATCGGAGTA
AF206880.1      TAGCATGGGATAACATCATAGGATTTCGGTCCTATTGTGTTGGCCTTCGGGATCGGAGTA
                ************************* **********  **********************
```

```
AY263903.1    ATGATTAACAGGGACAGTCGGGGGCATTCGTATTTCATAGTCAGAGGTGAAATTCTTGGA
AY263889.1    ATGATTAACAGGGACAGTCGGGGGCATTCGTATTTCATAGTCAGAGGTGAAATTCTTGGA
AY263902.1    ATGATTAACAGGGACAGTCGGGGGCATTCGTATTTCATAGTCAGAGGTGAAATTCTTGGA
AF132892.1    ATGATTAACAGGGACAGTCGGGGGCATTNGTATTTCATAGTCAGAGGTGAAATTCTTGGA
MF360746_Q    ATGATTAACAGGGACAGTCGGGGGCATTCGTATTTCATAGTCAGAGGTGAAATTCTTGGA
AF206931.1    ATGATTAACAGGGACAGTCGGGGGCATTCGTATTTCATAGTCAGAGGTGAAATTCTTGGA
AF206911.1    ATGATTAACAGGGACAGTCGGGGGCATTCGTATTTCATAGTCANAGGTTAAATTCTTGGA
JF317377.1    ATGATTAACAGGGACAGTCGGGGGCATTCGTATTTCATAGTCAGAGGTGAAATTCTTGGA
DQ886376.1    ATGATTAACAGGGACAGTCGGGGGCATTCGTATTTCATAGTCAGAGGTGAAATTCTTGGA
DQ886377.1    ATGATTAACAGGGACAGTCGGGGGCATTCGTATTTCATAGTCAGAGGTGAAATTCTTGGA
AY147111.1    ATGATTAACAGGGACAGTCGGGGGCATTCGTATTTCATAGTCAGAGGTGAAATTCTTGGA
U42515.1_C    ATGATTAACAGGAACAGTCGGGGGCATTCGTATTTCATAGTCAGAGGTGAAATTCTTGGA
AY263900.1    ATGATTAACAGGAACAGTCGGGGGCATTCGTATTTCATAGTCAGAGGTGAAATTCTTGGA
GU476453.1    ATGATTAACAGGAACAGTCGGGGGCATTCGTATTTCATAGTCAGAGGTGAAATTCTTGGA
AF206943.1    ATGATTAACAGGAACAGTCGGGGGCATTCGTATTTCATAGTCAGAGGTGAAATTCTTGGA
FJ469991.1    ATGATTAACAGGAACAGTCGGGGGCATTCGTATTTCATAGTCAGAGGTGAAATTCTTGGA
AF206880.1    ATGATTAACAGGAACAGTCGGGGGCATTCGTATTTCATAGTCAGAGGTGAAATTCTTGGA
              ************ *************** ************** **** ***********
AY263903.1    TTTATGAAAGACGAACAACTGCGAAAGCATTTGCCAAGGATGTTTTCATTAATCAAGAAC
AY263889.1    TTTATGAAAGACGAACAACTGCGAAAGCATTTGCCAAGGATGTTTTCATTAATCAAGAAC
AY263902.1    TTTATGAAAGACGAACAACTGCGAAAGCATTTGCCAAGGATGTTTTCATTAATCAAGAAC
AF132892.1    TTTATGAAAGASGAACAACTGCGAAAGCATTTGCCAAGGATGTTTTCATTAATCAAGAAC
MF360746_Q    TTTATGAAAGACGAACAACTGCGAAAGCATTTGCCAAGGATGTTTTCATTAATCAAGAAC
AF206931.1    TTTATGAAAGACGAACAACTGCGAAAGCATTTGCCAAGGATGTTTTCATTAATCAAGAAC
AF206911.1    TTTATGAAAGACGANCAACTGCGAAAGCATTTGCCAAGGATGTTTTCATTAATCAAGAAC
JF317377.1    TTTATGAAAGACGAACAACTGCGAAAGCATTTGCCAAGGATGTTTTCATTAATCAAGAAC
DQ886376.1    TTTATGAAAGACGAACAACTGCGAAAGCATTTGCCAAGGATGTTTTCATTAATCAAGAAC
DQ886377.1    TTTATGAAAGACGAACAACTGCGAAAGCATTTGCCAAGGATGTTTTCATTAATCAAGAAC
AY147111.1    TTTATGAAAGACGAACAACTGCGAAAGCATTTGCCAAGGATGTTTTCATTAATCAAGAAC
U42515.1_C    TTTATGAAAGACGAACAACTGCGAAAGCATTTGCCAAGGATGTTTTCATTAATCAAGAAC
AY263900.1    TTTATGAAAGACGAACAACTGCGAAAGCATTTGCCAAGGATGTTTTCATTAATCAAGAAC
GU476453.1    TTTATGAAAGACGAACAACTGCGAAAGCATTTGCCAAGGATGTTTTCATTAATCAAGAAC
AF206943.1    TTTATGAAAGACGAACAACTGCGAAAGCATTTGCCAAGGATGTTTTCATTAATCAAGAAC
FJ469991.1    TTTATGAAAGACGAACAACTGCGAAAGCATTTGCCAAGGATGTTTTCATTAATCAAGAAC
AF206880.1    TTTATGAAAGACGAACAACTGCGAAAGCATTTGCCAAGGATGTTTTCATTAATCAAGAAC
              *********** ** *********************************************
AY263903.1    GAAAGTTGGGGGCTCGAAGACGATCAGATACCGTCCTAGTCTCAACCATAAACGATGCCG
AY263889.1    GAAAGTTGGGGGCTCGAAGACGATCAGATACCGTCCTAGTCTCAACCATAAACGATGCCG
AY263902.1    GAAAGTTGGGGGCTCGAAGACGATCAGATACCGTCCTAGTCTCAACCATAAACGATGCCG
AF132892.1    GAAAGTTGGGGGCTCGAAGACGATCAGATACCGTCCTAGTCTCAACCATAAACGATGCCG
MF360746_Q    GAAAGTTGGGGGCTCGAAGACGATCAGATACCGTCCTAGTCTCAACCATAAACGATGCCG
AF206931.1    GAAAGTTGGGGGCTCGAAGACGATCAGATACCGTCCTAGTCTCAACCATAAACGATGCCG
```

**AF206911.1**	GAAAGTTGGGGGCTCGAAGACGATCAGATACCGTCCTAGTCTCAACCATAAACGATGCCG
**JF317377.1**	GAAAGTTGGGGGCTCGAAGACGATCAGATACCGTCCTAGTCTCAACCATAAACGATGCCG
**DQ886376.1**	GAAAGTTGGGGGCTCGAAGACGATCAGATACCGTCCTAGTCTCAACCATAAACGATGCCG
**DQ886377.1**	GAAAGTTGGGGGCTCGAAGACGATCAGATACCGTCCTAGTCTCAACCATAAACGATGCCG
**AY147111.1**	GAAAGTTGGGGGCTCGAAGACGATCAGATACCGTCCTAGTCTCAACCATAAACGATGCCG
**U42515.1_C**	GAAAGTTGGGGGCTCGAAGACGATCAGATACCGTCCTAGTCTCAACCATAAACGATGCCG
**AY263900.1**	GAAAGTTGGGGGCTCGAAGACGATCAGATACCGTCCTAGTCTCAACCATAAACGATGCCG
**GU476453.1**	GAAAGTTGGGGGCTCGAAGACGATCAGATACCGTCCTAGTCTCAACCATAAACGATGCCG
**AF206943.1**	GAAAGTTGGGGGCTCGAAGACGATCAGATACCGTCCTAGTCTCAACCATAAACGATGCCG
**FJ469991.1**	GAAAGTTGGGGGCTCGAAGACGATCAGATACCGTCCTAGTCTCAACCATAAACGATGCCG
**AF206880.1**	GAAAGTTGGGGGCTCGAAGACGATCAGATACCGTCCTAGTCTCAACCATAAACGATGCCG
	**
**AY263903.1**	ACCAGGGATCGGCGGATGTTACTTATAGGACTCCGCCGGCACCTTATGAGAAATCAAAGT
**AY263889.1**	ACCAGGGATCGGCGGATGTTACTTATAGGACTCCGCCGGCACCTTATGAGAAATCAAAGT
**AY263902.1**	ACCAGGGATCGGTGGATGTTACTTATAGGACTCCGCCGGCACATTATGAGAAATCAAAGT
**AF132892.1**	ACCAGGGATCGGCGGATGTTACTTATAGGACTCCGCCGGCACCTTATGAGAAATCAAAGT
**MF360746_Q**	ACCAGGGATTGGCGGATGTTACTTATAGGACTCCGCCAGCACCTTATGAGAAATCAAAGT
**AF206931.1**	ACCAGGGATTGGCGGATGTTGCTTTTAGGACTCCGCCAGCACCTTATGAGAAATCAAAGT
**AF206911.1**	ACCAGGGATCGGCGGATGTTGCTTATAGGACTCCGCCGGCACCTTATGAGAAATCAAAGT
**JF317377.1**	ACCAGGGATCGGCGGATGTTGCTTATAGGACTCCGCCGGCACCTTATGAGAAATCAAAGT
**DQ886376.1**	ACCAGGGATCGGCGGATGTTACTTTTAGGACTCCGCCGGCACCTTATGAGAAATCAAAGT
**DQ886377.1**	ACCAGGGATCGGCGGATGTTACTTTTAGGACTCCGCCGGCACCTTATGAGAAATCAAAGT
**AY147111.1**	ACCAGGGATCGGCGGATGTTACTTTTAGGACTCCGCCGGCACCTTATGAGAAATCAAAGT
**U42515.1_C**	ACCAGGGATCGGCGGATGTTGCTTTCAGGACTCCGCCGGCACCTTATGAGAAATCAAAGT
**AY263900.1**	ACCAGGGATCGGCGGATGTTGCTTTCAGGACTCCGCCGGCACCTTATGAGAAATCAAAGT
**GU476453.1**	ACCAGGGATCGGCGGATGTTGCTTTCAGGACTCCGCCGGCACCTTATGAGAAATCAAAGT
**AF206943.1**	ACCAGGGATCGGCGGATGTTGCTTTAAGGACTCCGCCGGCACCTTATGAGAAATCAAAGT
**FJ469991.1**	ACCAGGGATCGGCGGATGTTGCTTTAAGGACTCCGCCGGCACCTTATGAGAAATCAAAGT
**AF206880.1**	ACCAGGGATCGGCGGATGTTGCTTTAAGGACTCCGCCGGCACCTTATGAGAAATCAAAGT
	********* ** ******* *** *********** **** *****************
**AY263903.1**	CTTTGGGTTCCGGGGGGAGTATGGTCGCAAGGCTGAAACTTAAAGGAATTGACGGAAGGG
**AY263889.1**	CTTTGGGTTCCGGGGGGAGTATGGTCGCAAGGCTGAAACTTAAAGGAATTGACGGAAGGG
**AY263902.1**	CTTTGGGTTCCGGGGGGAGTATGGTCGCAAGGCTGAAACTTAAAGGAATTGACGGAAGGG
**AF132892.1**	CTTTGGGTTCCGGGGGGAGTATGGTCGCAAGGCTGAAACTTAAAGGAATTGACGGAAGGG
**MF360746_Q**	CTTTGGGTTCCGGGGGGAGTATGGTCGCAAGGCTGAAACTTAAAGGAATTGACGGAAGGG
**AF206931.1**	TTTTGGGTTCCGGGGGGAGTATGGTCGCAAGGCTGAAACTTAAAGGAATTGACGGAAGGG
**AF206911.1**	TTTTGGGTTCCGGGGGGAGTATGGTCGCAAGGCTGAAACTTAAAGGAATTGACGGAAGGG
**JF317377.1**	TTTTGGGTTCCGGGGGGAGTATGGTCGCAAGGCTGAAACTTAAAGGAATTGACGGAAGGG
**DQ886376.1**	TTTTGGGTTCCGGGGGGAGTATGGTCGCAAGGCTGAAACTTAAAGGAATTGACGGAAGGG
**DQ886377.1**	TTTTGGGTTCCGGGGGGAGTATGGTCGCAAGGCTGAAACTTAAAGGAATTGACGGAAGGG
**AY147111.1**	CTTTGGGTTCCGGGGGGAGTATGGTCGCAAGGCTGAAACTTAAAGGAATTGACGGAAGGG
**U42515.1_C**	CTTTGGGTTCCGGGGGGAGTATGGTCGCAAGGCTGAAACTTAAAGGAATTGACGGAAGGG

```
AY263900.1     CTTTGGGTTCCGGGGGGAGTATGGTCGCAAGGCTGAAACTTAAAGGAATTGACGGAAGGG
GU476453.1     CTTTGGGTTCCGGGGGGAGTATGGTCGCAAGGCTGAAACTTAAAGGAATTGACGGAAGGG
AF206943.1     CTTTGGGTTCCGGGGGGAGTATGGTCGCAAGGCTGAAACTTAAAGGAATTGACGGAAGGG
FJ469991.1     CTTTGGGTTCCGGGGGGAGTATGGTCGCAAGGCTGAAACTTAAAGGAATTGACGGAAGGG
AF206880.1     CTTTGGGTTCCGGGGGGAGTATGGTCGCAAGGCTGAAACTTAAAGGAATTGACGGAAGGG
                ***********************************************************
AY263903.1     CACCACCAGGAGTGGAGCCTGCGGCTTAATTTGACTCAACACGGGGAAACTTACCAGGTC
AY263889.1     CACCACCAGGAGTGGAGCCTGCGGCTTAATTTGACTCAACACGGGGAAACTTACCAGGTC
AY263902.1     CACCACCAGGAGTGGAGCCTGCGGCTTAATTTGACTCAACACGGGGAAACTTACCAGGTC
AF132892.1     CACCACCAGGAGTGGAGCCTGCGGCTTAATTTGACTCAACACGGGGAAACTTACCAGGTC
MF360746_Q     CACCACCAGGAGTGGAGCCTGCGGCTTAATTTGACTCAACACGGGGAAACTTACCAGGTC
AF206931.1     CACCACCAGGAGTGGAGCCTGCGGCTTAATTTGACTCAACACGGGGAAACTTACCAGGTC
AF206911.1     CACCACCAGGAGTGGAGCCTGCGGCTTAATTTGACTCAACACGGGGAAACTTACCAGGTC
JF317377.1     CACCACCAGGAGTGGAGCCTGCGGCTTAATTTGACTCAACACGGGGAAACTTACCAGGTC
DQ886376.1     CACCACCAGGAGTGGAGCCTGCGGCTTAATTTGACTCAACACGGGGAAACTTACCAGGTC
DQ886377.1     CACCACCAGGAGTGGAGCCTGCGGCTTAATTTGACTCAACACGGGGAAACTTACCAGGTC
AY147111.1     CACCACCAGGAGTGGAGCCTGCGGCTTAATTTGACTCAACACGGGGAAACTTACCAGGTC
U42515.1_C     CACCACCAGGAGTGGAGCCTGCGGCTTAATTTGACTCAACACGGGGAAACTTACCAGGTC
AY263900.1     CACCACCAGGAGTGGAGCCTGCGGCTTAATTTGACTCAACACGGGGAAACTTACCAGGTC
GU476453.1     CACCACCAGGAGTGGAGCCTGCGGCTTAATTTGACTCAACACGGGGAAACTTACCAGGTC
AF206943.1     CACCACCAGGAGTGGAGCCTGCGGCTTAATTTGACTCAACACGGGGAAACTTACCAGGTC
FJ469991.1     CACCACCAGGAGTGGAGCCTGCGGCTTAATTTGACTCAACACGGGGAAACTTACCAGGTC
AF206880.1     CACCACCAGGAGTGGAGCCTGCGGCTTAATTTGACTCAACACGGGGAAACTTACCAGGTC
               ************************************************************
AY263903.1     CAGACATAGTAAGGATTGACAGACTGAGAGCTCTTTTTTGATTCTATGGGTGGTGGGGCA
AY263889.1     CAGACATAGTAAGGATTGACAGACTGAGAGCTCTTTCTTGATTTTATGGGTGGTGGTGCA
AY263902.1     CAGACATAGTAAGGATTGACAGACTGAGAGCTCTTTCTTGATTNTATGGGTGGTGGTGCA
AF132892.1     CAGACATAGTAAGGATTGACAGACTGAGAGCTCTTTCTTGATTCTATGGGTGGTGGTGCA
MF360746_Q     CAGACATAGTAAGGATTGACAGACTGAGAGCTCTTTCTTGATTCTATGGGTGGTGGTGCA
AF206931.1     CAGACATAGTAAGGATTGACAGATTGAGAGCTCTTTCTTGATTCTATGGGTGGTGGTGCA
AF206911.1     CAGACATAGCAAGGATTGACAGACTGAGAGCTCTTTCTTGATTCTATGGGTGGTGGTGCA
JF317377.1     CAGACATAGTAAGGATTGACAGACTGAGAGCTCTTTCTTGATTCTATGGGTGGTGGTGCA
DQ886376.1     CAGACATAGTAAGGATTGACAGACTGAGAGCTCTTTCTTGATTCTATGGGTGGTGGTGCA
DQ886377.1     CAGACATAGTAAGGATTGACAGACTGAGAGCTCTTTCTTGATTCTATGGGTGGTGGTGCA
AY147111.1     CAGACATAGTAAGGATTGACAGACTGAGAGCTCTTTCTTGATTCTATGGGTGGTGGTGCA
U42515.1_C     CAGACATAGTAAGGATTGACAGACTGAGAGCTCTTTCTTGATTCTATGGGTGGTGGTGCA
AY263900.1     CAGACATAGTAAGGATTGACAGACTGAGAGCTCTTTCTTGATTCTATGGGTGGTGGTGCA
GU476453.1     CAGACATAGTAAGGATTGACAGACTGAGAGCTCTTTCTTGATTCTATGGGTGGTGGTGCA
AF206943.1     CAGACATAGTAAGGATTGACAGACTGAGAGCTCTTTCTTGATTCTATGGGTGGTGGTNCA
FJ469991.1     CAGACATAGTAAGGATTGACAGACTGAGAGCTCTTTCTTGATTCTATGGGTGGTGGTGCA
AF206880.1     CAGACATAGTAAGGATTGACAGACTGAGAGCTCTTTCTTGATTCTATGGGTGGTGGTGCA
               ********* ************* ************ ****** ************  **
```

```
AY263903.1      TGGCCGTTTTTAGTTGGTGGAGTAATTTGTCTGGTTAATTCCGTTAACGAACGAGACCTC
AY263889.1      TGGCCGTTCTTAGTTGGTGGAGTGATTTGTCTGGTTAATTCCGTTAACGAACGAGACCTC
AY263902.1      TGGCCGTTCTTAGTTGGTGGAGTGATTTGTATGGTTAATTCCGTTAAGGAACGAGACCTC
AF132892.1      TGGCCGTTCTTAGTTGGTGGAGTGATTTGTCTGGTTAATTCCGTTAACGAACGAGACCTC
MF360746_Q      TGGCCGTTCTTAGTTGGTGGAGTGATTTGTCTGGTTAATTCCGTTAACGAACGAGACCTC
AF206931.1      TGGCCGTTCTTAGTTGGTGGAGCGATTTGTCTGGTTAATTCCGTTAACGAACGAGACCTC
AF206911.1      TGGCCGTTCTTAGTTGGTGGAGCGATTTGTCTGGTTAATTCCGTTAACGAACGAGACCTC
JF317377.1      TGGCCGTTCTTAGTTGGTGGAGCGATTTGTCTGGTTAATTCCGTTAACGAACGAGACCTC
DQ886376.1      TGGCCGTTCTTAGTTGGTGGAGCGATTTGTCTGGTTAATTCCGTTAACGAACGAGACCTC
DQ886377.1      TGGCCGTTCTTAGTTGGTGGAGCGATTTGTCTGGTTAATTCCGTTAACGAACGAGACCTC
AY147111.1      TGGCCGTTCTTAGTTGGTGGAGCGATTTGTCTGGTTAATTCCGTTAACGAACGAGACCTC
U42515.1_C      TGGCCGTTCTTAGTTGGTGGAGCGATTTGTCTGGTTAATTCCGTTAACGAACGAGACCTC
AY263900.1      TGGCCGTTCTTAGTTGGTGGAGCGATTTGTCTGGTTAATTCCGTTAACGAACGAGACCTC
GU476453.1      TGGCCGTTCTTAGTTGGTGGAGCGATTTGTCTGGTTAATTCCGTTAACGAACGAGACCTC
AF206943.1      TGNCCGTTCTTAGTTGGTGGAGCGATTTGTCTGGTTAATTCCGTTAACGAACGAGACCTC
FJ469991.1      TGGCCGTTCTTAGTTGGTGGAGCGATTTGTCTGGTTAATTCCGTTAACGAACGAGACCTC
AF206880.1      TGGCCGTTCTTAGTTGGTGGAGCGATTTGTCTGGTTAATTCCGTTAACGAACGAGACCTC
                ** ***** *************  ****** **************** ************

AY263903.1      AGCGTGTTAACTAGCTATGCGGAGGTGACCCTTCGCGGCCAGCTTCTTAGAGGGAATATG
AY263889.1      AGCCTGCTAACTAGCTATGCGGAGGTGACCCTCCGCGGCCAGCTTNTTAGAGGAGCTATG
AY263902.1      AGCCTGCTAACTAGCTATGCGGAGGTGACCCTCCGCGGCCAGCTTCTTAGAGGGACTATG
AF132892.1      AGCCTGCTAACTAGCTATGCGGAGGTGACCCTCCGCGGCCAGCTTCTTAGAGGGACTATG
MF360746_Q      AGCCTGCTAACTAGCTATGCGGAGGTGACCCTCCGCGGCCAGCTTCTTAGAGGGACTATG
AF206931.1      AGCCTGCTAACTAGCTATGCGGAGGATTTCCTCCGCGGCCAGCTTCTTAGAGGGACTATG
AF206911.1      AGCCTGCTAACTAGCTATGCGGAGGATACCCTTCGCGGCCAGCTTCTTAGAGGGACTATG
JF317377.1      AGCCTGCTAACTAGCTATGCGGAGGTATCCCTCCGCAGCTAGCTTCTTAGAGGGACTATG
DQ886376.1      AGCCTGCTAACTAGCTATGCGGAGGAT-CCCTCCGCGGCCAGCTTCTTAGAGGGACTATG
DQ886377.1      AGCCTGCTAACTAGCTATGCGGAGGAT-CCCTCCGCGGCCAGCTTCTTAGAGGGACTATG
AY147111.1      AGCCTGCTAAATAGCTATGCGGAGGAGACCCTCCGTGGCCAGCTTCTTAGAGGGACTATG
U42515.1_C      AGCCTGTTAACTAGCTATGCGGAGGTGACCCTCCGCGGCCAGCTTCTTAGAGGGACCACG
AY263900.1      AGCCTGTTAACTAGCTATGCGGAGGTGACCCTCCGCGGCCAGCTTCTTAGAGGGACTATG
GU476453.1      AGCCTGTTAACTAGCTATGCGGAGGATCCCCTCCGCGGCCAGCTTCTTAGAGGGACTATG
AF206943.1      AGCCTGCTAACTAGCTATGCGGAGGTGACCTTCCGCGGCCAGCTTCTTAGAGGGACTATG
FJ469991.1      AGCCTGCTAACTAGCTATGCGGAGGTGACCTTCCGCGGCCAGCTTCTTAGAGGGACTATG
AF206880.1      AGCCTGCTAACTAGCTATGCGGAGGTGACCTTCCGCGGCCAGCTTCTTAGAGGGACTATG
                *** ** *** **************    * * **  **  ***** *******   * *

AY263903.1      GCCGCTTAGGCCAAGGAAGTTTGAGGCAATAACAGGTCTGTGATGCCCTTAGATGTTTTG
AY263889.1      GCCGCTTAGGCCAAGGAAGTTTGAGGCAATAACAGGTCTGTGATGCCCTTAGATGTTTTG
AY263902.1      GCCGCTTAGGCCAAGGAAGTTTGAGGCAATAACAGGTCTGTGATGCCCTTAGATGTTCTG
AF132892.1      GCCGCTTAGGCCAAGGAAGTTTGAGGCAATAACAGGTCTGTGATGCCCTTAGATGTTCTG
MF360746_Q      GCCGCTTAGGCCAAGGAAGTTTGAGGCAATAACAGGTCTGTGATGCCCTTAGATGTTCTG
AF206931.1      GCCGCTTAGGCCAAGGAAGTTTGAGGCAATAACAGGTCTGTGATGCCCTTAGATGTTCTG
```

```
AF206911.1      GCCGCTTAGGCCAAGGAAGTTTGAGGCAATAACAGGTCTGTGATGCCCTTAGATGTTCTG
JF317377.1      GCCGCTTAGGCCAAGGAAGTTTGAGGCAATAACAGGTCTGTGATGCCCTTAGATGTTCTG
DQ886376.1      GCCGCTTAGGCCAAGGAAGTTTGAGGCAATAACAGGTCTGTGATGCCCTTAGATGTTCTG
DQ886377.1      GCCGCTTAGGCCAAGGAAGTTTGAGGCAATAACAGGTCTGTGATGCCCTTAGATGTTCTG
AY147111.1      GCCTTTTAGGCCAAGGAAGTTTGAGGCAATAACAGGTCTGTGATGCCCTTAGATGTTCTG
U42515.1_C      GCCGTTCAGACCGCGGAAGTTTGAGTCAATAACAGGTCTGTGATGCCCTTAGATGTTCTG
AY263900.1      GCCGCTTAGGCCACGGAAGTTTGAGGCAATAACAGGTCTGTGATGCCCTTAGATGTTCTG
GU476453.1      GCCGCTTAGGCCACGGAAGTTTGAGGCAATAACAGGTCTGTGATGCCCTTAGATGTTCTG
AF206943.1      GCCGCTTAGGCCAAGGAAGTTTGAGGCAATAACAGGTCTGTGATGCCCTTAGATGTTCTG
FJ469991.1      GCCGCTTAGGCCAAGGAAGTTTGAGGCAATAACAGGTCTGTGATGCCCTTAGATGTTCTG
AF206880.1      GCCGCTTAGGCCAAGGAAGTTTGAGGCAATAACAGGTCTGTGATGCCCTTAGATGTTCTG
                ***  * ** **  *********** ******************************* **
AY263903.1      GGCCGCACGCGCGATACACTGATGTATTCAACGAGTTTATAGCCTTGGCCGGCAGGCCCG
AY263889.1      GGCCGCACGCGCGNTACACTGATGTATTCAACGAGTTTATAGCCNNGGCCGACAGGCCCG
AY263902.1      GGCCGCACGCGCGCTACACTAATGTATTCAACGAGTTTATAGCCTTGGCCGACAGGCCCG
AF132892.1      GGCCGCACGCGCGCTACACTGATGTATTCAACGAGTTTATAGCCTTGGCCGACAGGCCCG
MF360746_Q      GGCCGCACGCGCGCTACACTGATGTATTCAACGAGTTTATAGCCTTGGCCGACAGGCCCG
AF206931.1      GGCCGCACGCGCGCTACACTGATGTATTCAACGAGTCTATAGCCTTGGCCGACAGGCCCG
AF206911.1      GGCCGCACGCGCGCTACACTGATGTATTCAACGAGTCTATAGCCTTGGCCGACAGGCCCG
JF317377.1      GGCCGCACGCGCGCTACACTGATGTATTCAACGAGTCTATAGCCTTGGCCGACAGGCCCG
DQ886376.1      GGCCGCACGCGCGCTACACTGATGTATTCAACGAGTCTATAGCCTTGGCCGACAGGCCCG
DQ886377.1      GGCCGCACGCGCGCTACACTGATGTATTCAACGAGTCTATAGCCTTGGCCGACAGGCCCG
AY147111.1      GGCCGCACGCGCGCTACACTGATGTATTCAACGAGTTTATAGCCTTGGCCGACAGGCCCG
U42515.1_C      GGCCGCACGCGCGCTACACTGATGTATTCAACGAGTCTATAGCCTTGGCCGACAGGCCCG
AY263900.1      GGCCGCACGCGCGCTACACTGATGTATTCAACGAGTTTATAGCCTTGGCCGACAGGCCCG
GU476453.1      GGCCGCACGCGCGCTACACTGATGTATTCAACGAGTTTATAGCCTTGGCCGACAGGCCCG
AF206943.1      GGCCGCACGCGCGCTACACTGATGTATTCAACGAGTTTATAGCCTTGGCCGACAGGCCCG
FJ469991.1      GGCCGCACGCGCGCTACACTGATGTATTCAACGAGTTTATAGCCTTGGCCGACAGGCCCG
AF206880.1      GGCCGCACGCGCGCTACACTGATGTATTCAACGAGTTTATAGCCTTGGCCGACAGGCCCG
                ************* ****** *************** *******  ***** ********
AY263903.1      GGTAATCTTTGAAATTTCATCGTGATGGGGATAGATCATTGCAATTGTTGGTCTTCAACG
AY263889.1      GGTAATCTTTGAAATTTCNTCGTGATGGGGATAGATCATTGCAATTGTTGGTCTTCAACG
AY263902.1      GGTAATCTTTGAAATTTCATCGTAATGGGGATAGATCATTGCAATTGTTGGTCTTCAACG
AF132892.1      GGTAATCTTTGAAATTTCATCGTGATGGGGATAGATCATTGCAATTGTTGGTCTTCAACG
MF360746_Q      GGTAATCTTTGAAATTTCATCGTGATGGGGATAGATCATTGCAATTGTTGGTCTTCAACG
AF206931.1      GGTAATCTTTGAAATTTCATCGTGATGGGGATAGATCATTGCAATTGTTGGTCTTCAACG
AF206911.1      GGTAATCTTTGAAATTTCATCGTGATGGGGATAGATCATTGCAATTGTTGGTCTTCAACG
JF317377.1      GGTAATCTTTGAAATTTCATCGTGATGGGGATAGATCATTGCAATTGTTGGTCTTCAACG
DQ886376.1      GGTAATCTTTGAAATTTCATCGTGATGGGGATAGATCATTGCAATTGTTGGTCTTCAACG
DQ886377.1      GGTAATCTTTGAAATTTCATCGTGATGGGGATAGATCATTGCAATTGTTGGTCTTCAACG
AY147111.1      GGTAATCTTTGAAATTTCATCGTGATGGGGATAGATCATTGCAATTGTTGGTCTTCAACG
U42515.1_C      GGTAATCTTTGAAATTTCATCGTGATGGGGATAGATCATTGCAATTGTTGGTCTTCAACG
```

```
AY263900.1    GGTAATCTTTGAAATTTCATCGTGATGGGGATAGATCATTGCAATTGTTGGTCTTAAACG
GU476453.1    GGTAATCTTTGAAATTTCATCGTGATGGGGATAGATCATTGCAATTGTTGGTCTTCAACG
AF206943.1    GGTAATCTTTGAAATTTCATCGTGATGGGGATAGATCATTGCAATTGTTGGTCTTCAACG
FJ469991.1    GGTAATCTTTGAAATTTCATCGTGATGGGGATAGATCATTGCAATTGTTGGTCTTAAACA
AF206880.1    GGTAATCTTTGAAATTTCATCGTGATGGGGATAGATCATTGCAATTGTTGGTCTTCAACG
              ****************** **** ******************************* *** 
AY263903.1    AGGAATT-CCTAGTAAGCGCGAGTCATCAGCTCGCGTTGACTACGTCCC-TGCCCTTTGT
AY263889.1    AGGAATT-CCTAGTAAGCGCGAGTCATCAGCTCGCGTTGACTACGTCCC-TGCCCTTTGT
AY263902.1    AGGAATT-CCTAGTAAGCGCGAGTCATCAGCTCGCGTTGACTACGTCCC-TGCCCTTTGT
AF132892.1    ARGAATT-CCTAGTAAGCGCGAGTCATCAGCTCGCGTTGACTACGTCCC-TGCCCTTTGT
MF360746_Q    AGGAATT-CCTAGTAAGCGCGAGTCATCAGCTCGCGTTGACTACGTCCC-TGCCCTTTGT
AF206931.1    AGGAATT-CCTAGTAAGCGCGAGTCATCAGCTCGCGTTGACTACGTCCC-TGCCCTTTGT
AF206911.1    AGGAATT-CCTAGTAAGCGCGAGTCATCAGCTCGCGTTGACTACGTCCC-TGCCCTTTGT
JF317377.1    AGGAATT-CCTAGTAAGCGCGAGTCATCAGCTCGCGTTGACTACGTCCC-TGCCCTTTGT
DQ886376.1    AGGAATT-CCTAGTAAGCGCGAGTCATCAGCTCGCGTTGACTACGTCCC-TGCCCTTTGT
DQ886377.1    AGGAATT-CCTAGTAAGCGCGAGTCATCAGCTCGCGTTGACTACGTCCC-TGCCCTTTGT
AY147111.1    AGGAATT-CCTAGTAAGCGCGAGTCATCAGCTCGCGTTGACTACGTCCC-TGCCCTTTGT
U42515.1_C    AGGAATT-CCTAGTAAGCGCGAGTCATCAGCTCGCGTTGACTACGTCCC-TGCCCTTTGT
AY263900.1    AGGAATT-CCTAGTAAGCGCGAGTCATCAGCTCGCGTTGACTACGTCCC-TGCCCTTTGT
GU476453.1    AGGAATT-CCTAGTAAGCGCGAGTCATCAGCTCGCGTTGACTACGTCCC-TGCCCTTTGT
AF206943.1    AGGAATT-CCTAGTAAGCGCGAGTCATCAGCTCGCGTTGACTACGTCCC-TGCCCTTTGT
FJ469991.1    GAGAATTTCCTAGTAAGCGCGAGTCATCAGCTCGCGTTGACTACGTCCCCTGCCCTTTG-
AF206880.1    AGGAATT-CCTAGTAAGCGCGAGTCATCAGCTCGCGTTGACTACGTCCC-TGCCCTTTGT
                ***** ***************************************** ********* 
AY263903.1    ACACACCGCCCGTCGCTCCTACCGATTGAATGGTC-------------------------
AY263889.1    ACACACCGCCCGTCGCTCCTACCGATTGAATGGTCCGGTGAAGTGTTCGGATCGCGGCGA
AY263902.1    ACACACCGCCCGTCGCTCCTACCGATTGAATGGTC-------------------------
AF132892.1    ACACACCGCCCGTCGCTCCTACCGATTGAATGGTCCGGTGAAGTGTTCGGATCGCGGCGA
MF360746_Q    ACACACCGCCCGTCGCTCCTACCGATTGAATGGTCCGGTGAAGTGTTCGGATCGCGGCGA
AF206931.1    ACACACCGCCCGTCGCTCCTACCGATTGAATGGTCCGGTGAAGTGTTCGGATCGAGGCGA
AF206911.1    ACACACCGCCCGTCGCTCCTACCGATTGAATGGTCCGGTGAAGTGTTCGGATCGAGGCGA
JF317377.1    ACACACCGCCCGTCGCTCCTACCGATTGAATGGTCCGGTGAAATGTTCGGATCGCGGCGA
DQ886376.1    ACACACCGCCCGTCGCTCCTACCGATTGAATGGTCCGGTGAAGTGTTCGGATCGCGGCGA
DQ886377.1    ACACACCGCCCGTCGCTCCTACCGATTGAATGGTCCGGTGAAGTGTTCGGATCGCGGCGA
AY147111.1    ACACACCGCCCGTCGCTCCTACCGATTGAATGGTCCGGTGAAGTGTTCG-----------
U42515.1_C    ACACACCGCCCGTCGCTCCTACCGATTGAATGGTCCGGTGAAGTGTTCGGATCGCGGCGA
AY263900.1    ACACACCGCCCGTCGCTCCTACCGATTGAATGGTCCGGTGAAGTGTTCGGATCGCGGCGA
GU476453.1    ACACACCGCCCGTCGCTCCTACCGATTGAATGGTCCGGTGAAGTGTTCG-----------
AF206943.1    ACACACCGCCCGTCGCTCCTACCGATTGAATGGTCCGGTGAAGTGTTCGGATCGAGGCGA
FJ469991.1    ------------------------------------------------------------
AF206880.1    ACACACCGCCCGTCGCTCCTACCGATTGAATGGTCCGGTGAAGTGTTCGGATCGCGGCGA
```

```
AY263903.1     ------------------------------------------------------------
AY263889.1     CG-GGGCGGTTCGCTGCCGGCG--------------------------------------
AY263902.1     ------------------------------------------------------------
AF132892.1     CGTGGGCGGTTCGCTGCCGGCGACNTCGCGAGAAGTCCACTGAACCTTATCATTTAGAAG
MF360746_Q     CGTGGGCGGTTCGCTGCCGGCGACGTCGCGAGAAGTCCACTGAACCTTATCATTTAGAGG
AF206931.1     CGTGGGCGGTTCGCTGCCCGCGACGTAGCGAGAAGTCCACTGAACCTTATCATTTAGAGG
AF206911.1     CATGGGCGGTCCGCTGCCCGTGACGTCGCGAGAAGTCCACTGAACCTTATCATTGAGAGG
JF317377.1     CGTGGGCGGTTCGCTGCCCGCGACGTCGCGAGAAGTCCATTGAACCTTATCATTTAG---
DQ886376.1     CGTGGGTGGTTCGCTGCCCGCGACGTCGCGAGAAGTCCACTGAACCTTATCATT------
DQ886377.1     CGTGGGTGGTTCGCTGCCCGCGACGTCGCGAGAAGTCCACTGAACCTTATCATT------
AY147111.1     ------------------------------------------------------------
U42515.1_C     CGTGGGCGGTTCGCTGCCGGCGACGTCGCGAGAAGTCCACTGAACCTTATCATTTAG---
AY263900.1     CGTGGGCGGTTCG-----------------------------------------------
GU476453.1     ------------------------------------------------------------
AF206943.1     TGTGGGCGGTTCGCTGCCGGCAACGTCGCGAGAAGTCCACTGNACCTTATCATNTAGNGG
FJ469991.1     ------------------------------------------------------------
AF206880.1     TGTGGGCGGTCCGCTGCCGGCAACGTCGCGAGAAGTCCACTGAACCTTATCATTTAGAGG

AY263903.1     -------------------
AY263889.1     -------------------
AY263902.1     -------------------
AF132892.1     AAGGA--------------
MF360746_Q     AAGGAGAAGTCGTAACAAG
AF206931.1     AAGGAGAA-----------
AF206911.1     AAGGAGC------------
JF317377.1     -------------------
DQ886376.1     -------------------
DQ886377.1     -------------------
AY147111.1     -------------------
U42515.1_C     -------------------
AY263900.1     -------------------
GU476453.1     -------------------
AF206943.1     A------------------
FJ469991.1     -------------------
AF206880.1     AAGGA--------------
```

Figure S2. DNA sequence alignment of the 18S rDNA segments used for phylogenetic analyses (tree presented in Figure S1). The 18S rDNA segment identified for the first time in this study is highlighted in yellow. Nucleotide identity in given positions is denoted by the asterisk * at the bottom of the alignment.

(a)

5′AGTGTTTCTTATGGACAATGGGATTATTGTTTCAGCCACAATTTGGATATTTTAGGAGAAC
AATAGCAAAGTTCTTTGCACTAATAACAGTAATAGATGATATTTATGATGCATATGGCACTT
TGGAAGAACTGGAGTGCTTCACAAATGCTATCGAGAGATGGGA3′

(b)

```
BAD98243.1      MATELLCLHRPISLTHKLFRNPLP--------KVIQATPLTLKLRCSVSTENVSFTETET
AAQ16588.1      MATELLCLHRPISLTHKLFRNPLP--------KVIQATPLTLKLRCSVSTENVSFSETET
CAL69918.1      MATELLCLHRPISLTHKLFRNPLP--------KVIQATPLTLKLRCSVSTENVSFTETET
AEK70970.1      MATELLCLHRPISLTPKLFRNPLP--------KVILATPLTLKLRCSVSTENVSFTETET
AAQ84170.1      MATNLLCLSNKLSSPTPTPSTRFPQSKNFITQKTSLANPKPWRVICATSSQFT--QITEH
CUR52979.1      MATNPSCLSTPFLSSTPALSTRFPLSENFT-QKTSLVNPKPWPLISAVSSQFS--QIAED
AEK70968.1      ------------------------------------------------------------
AEK70969.1      ------------------------------------------------------------
BAS30551.1      --MIMATDQLLCMNPGCFFAHKLG---------TSQSARRPLQGIASTTSRPSQLVRCSA
BAS30550.1      MIHIMAT-ELLYMSPGCFFAHKLS---------TSQSARRPLQGIASTTSRPSQMVRCSA
AAP40638.1      ------------MALRLLSTPHLP---------QLCSRRVSGRVHCSASTQVSDAQ----
BAF02831.1      ------------MALRLLFTPHLP---------VLSSRRANGRVRCSASTQISDPQ----
UQP00000.1      ------------------------------------------------------------
BAS30549.1      -MELSLAASLANCNFTRLLPSKTS---------ISLVASRRASIRPAVLCMAISETSTES

BAD98243.1      EARRSANYEPNSWDYDYLLSSDTD------ESIEVYKDKAKKLEAEVRREINNEKAEFLT
AAQ16588.1      ETRRSANYEPNSWDYDYLLSSDTD------ESIEVHKDKAKKLEAEVRREINNEKAEFLT
CAL69918.1      ETRRSANYEPNSWDYDYLLSSDTD------ESIEVYKDKAKKLEAEVRREINNEKAEFLT
AEK70970.1      ETRRSANYEPNSWDYDYLLSSDTD------ESIEVYKDKAKKLEAEVRREINNEKAEFLT
AAQ84170.1      NSRRSANYQPNLWNFEFLQSLEND------LKVEKLEEKATKLEEEVRCMINRVDTQPLS
CUR52979.1      NSRRSANYHPNLWDFEFLQSLEND------SKMEKLEEKATKLEEEVRNMMNEAKTEALS
AEK70968.1      ---RSANYQPNLWNFEFLQSQEYD------LMVETLQERATKLEEEVRRLINRVDIEPLK
AEK70969.1      --RRSGNYQPNLWNFDFLQSQKND------LKEEMLQERAGKLEEEVRGLINEVDTEPLS
BAS30551.1      SQRLSANFQPSSWSYDFLQSLEAN------YHVETYKEKVEKLEKEVKRMINKEDASLMT
BAS30550.1      DQRLSANYQPSSWSYDFLQSLEAN------YHVETYKEKVEKLEKEVRHIINKEDASLMT
AAP40638.1      GGRRSANYQPSVWTYNYLQSLVADDIRRSRREVEQEREKAQILEEDVRGALNDGNAEPMA
BAF02831.1      EGRRSANYQPSVWTYNYLQSIVAGEGRQSRREVEQQKEKVQILEEEVRGALNDEKAETFT
UQP00000.1      ------------------------------------------------------------
BAS30549.1      VVRRSANFQPAIWHYDFIQSLRSD------YTEESCSQRIDKLKGEVRMMLQKA-VDPLE

BAD98243.1      LLELIDNVQRLGLGYRFESDIRGALDRFVS--SGGFDAVTKTSLHGTALSFRLLRQHGF-
AAQ16588.1      LLELIDNVQRLGLGYRFESDIRRALDRFVS--SGGFDGVTKTSLHGTALSFRLLRQHGF-
CAL69918.1      LPELIDNVQRLGLGYRFESDIRRALDRFVS--SGGFDAVTKTSLHATALSFRLLRQHGF-
AEK70970.1      LLELIDNVQRLGLGYRFESDIRRALDRFVS--SGGFDAVTKTSLHATALSFRFLRQHGF-
AAQ84170.1      LLELIDDVQRLGLTYKFEKDIIKALENIVL--LDENK-KNKSDLHATALSFRLLRQHGF-
CUR52979.1      LLELIDDVQRLGLTYKFEKDIIKALEKIVP--LDE------SGLHVTSLSFRILRQHGF-
```

```
AEK70968.1      LLELVDNVQRLGLTYKFEDDINKALERIVS--LDE---REKSGLHATALIFRLLRQHGF-
AEK70969.1      LLELIDNVERLGLTYKFQEDINKALGRIVS--SDIN----KSGLHAAALTFRLLRQHGF-
BAS30551.1      TLELIDDIQRLGLGYRFEEEIREALERISS--LEGFNSGIEKSLHATALGFRLLRQHGFN
BAS30550.1      TLELIDDIQRLGLGYRFEEDIRKALERISS--LEGFDSGIEKSLHAAALGFRLLRQHGFN
AAP40638.1      IFALVDDIQRLGLGRYFEEDISKALRRCLS--QYAVTGSLQKSLHGTALSFRVLRQHGF-
BAF02831.1      IFATVDDIQRLGLGDHFEEDISNALRRCVS--KGAVFMSLQKSLHGTALGFRLLRQHGY-
UQP00000.1      ------------------------------------------------------------
BAS30549.1      RLELIDVLQRLGLSYHFDEEIQKILESIYDANYGGKIPSNKENIYATALEFRLLRQLGY-

BAD98243.1      EVSQEAFSGFKDQNGNFLENLKEDIKAILSLYEASFLALEGENILDEAKVFAISHLKELS
AAQ16588.1      EVSQEAFSGFKDQNGNFLENLKEDIKAILSLYEASFLALEGENILDEAKVFAISHLKELS
CAL69918.1      EVSQEAFSGFKDQNGNFLKNLKEDIKAILSLYEASFLALEGENILDEAKVFAISHLKELS
AEK70970.1      EVSQEAFGGFKDQNGNFLENLKEDIKAILSLYEASFLALEGENILDEAKVFAISHLKELS
AAQ84170.1      EVSQDVFERFKDKEGGFSGELKGDVQGLLSLYEASYLGFEGENLLEEARTFSITHLKNNL
CUR52979.1      EVSQDVFKRFKDKEGGFCAELKDDVQGLLSLYEASYLGFEGESLLDEARAFSITHLKNNL
AEK70968.1      EVSQDVFESTRDKEGRFKAEIKGDVQGLLSLYEASYLGFEGENLLDEAREFSMTHLKN-L
AEK70969.1      QISQDVFEKFKDKEGRFSAEIKGDVQGLLSLYEASYLGFEGENVLEEARAFSTTHLRN-I
BAS30551.1      YVSQDISKIIKDQNGSFKESLSKDVKGMLSLYEASHLAFQGESPWGEAREFTRTHLNDLI
BAS30550.1      YVSQDIFKIIKDQNGSIKESLSKDVKGMLSLYEASHLAFQGESLWDEAREFTRTHLNDLI
AAP40638.1      EVSQDVFKIFMDESGSFMKTLGGDVQGMLSLYEASHLAFEEEDILHKAKTFAIKHLENLN
BAF02831.1      EVSQDVFKIFLDESGSFVKTLGGDVQGVLSLYEASHLAFEEEHILHKARSFAIKHLENLN
UQP00000.1      ------------------------------------------------------------
BAS30549.1      GVPQEIFNSFRNEQGNFKASLCDDIKGILCLYEASFLLVEGETILEETRDFTTKQLKEYI

BAD98243.1      EEKIGK-ELAEQVNHALELPLHRRTQRLEAVWSIEAYRKKEDANQVLLELAILDYNMIQS
AAQ16588.1      EEKIGK-ELAEQVSHALELPLHRRTQRLEAVWSIEAYRKKEDANQVLLELAILDYNMIQS
CAL69918.1      EEKIGK-DLAEQVNHALELPLHRRTQRLEAVWSIEAYRKKEDADQVLLELAILDYNMIQS
AEK70970.1      EEKIGK-DLAEQVNHALELPLHRRTQRLEAVWSIEAYRKKEDANQVLLELAILDYNMIQS
AAQ84170.1      KEGINT-KVAEQVSHALELPYHQRLHRLEARWFLDKYEPKEPHHQLLLELAKLDFNMVQT
CUR52979.1      NKGINT-KVAQQVSHALELPYHRRLHRLEARWLLDKYEPKEPHHHLLHELAKLDFNLVQS
AEK70968.1      NEGVVTPKLAEQINHALELPYHRRFQRLEARWFIENYEVKEPHDRLLVELAKLDFNMVQS
AEK70969.1      KQGVST-KMAEQISHALELPYHRRLQRLEARRFIDKFEIKEPQDRLLLELAKLDFNMVQT
BAS30551.1      RSGDLSKDVAEEIRHALELPLHQRMNRLEARRYIEGYAKRSDANRVLLEFAKWDFNMVQS
BAS30550.1      RSDDLSKDVAEEIRHALELPLHQRMNRLEARRYIEGYAKRSDANRVLLEFAKWDFNMVQS
AAP40638.1      --HDIDQDLQDHVNHELELPLHRRMPLLEARRFIEAYSRRSNVNPRILELAVMKFNSSQL
BAF02831.1      --SDVDKDLQDQVKHELELPLHRRMPLLEARRSIEAYSRRGYTNPQILELALTDFNVSQS
UQP00000.1      ------------------------------------------------------------
BAS30549.1      KQSTDE-NLTDLVSHALEVPLHWRMLRLETRRFIDVYRSREDANPILLELATLDFNLVQT

BAD98243.1      VYQRDLRETSRWWRRVGLATKLHFARDRLIESFYWAVGVAFEPQYSDCRNSVAKMFSFVT
AAQ16588.1      VYQRDLRETSRWWRRVGLATKLHFARDRLIESFYWAVGVAFEPQYSDCRNSVAKMFSFVT
CAL69918.1      VYQRDLRETSRWWRRVGLATKLHFARDRLIESFYWAVGVAFEPQYSDCRNSVAKMFSFVT
```

```
AEK70970.1      VYQRDLRETSRWWRRVGLATKLHFARDRLIESFYWAVGVAFEPQYSDCRNSVAKMFSFVT
AAQ84170.1      LHQKELQDLSRWWTEMGLASKLDFVRDRLMEVYFWALGMAPDPQFGECRKAVTKMFGLVT
CUR52979.1      LYQKELRELSLWWREIGLTSKLDFVRDRLMEVYFWALGMAPDPQFSECRKVVTKMFGLVT
AEK70968.1      LQKKEVGELSRWWKEIGLTSKLDFVRDRLVEVYFWASGMAPDPQLSECRKAVTKMFGLVT
AEK70969.1      LQQKELRDLSRWWKEIGLARKMEFVRDRLMEVYFWAVGMAPDPLLSDCRKAIAKMFGLVT
BAS30551.1      TLQNDLKDLSRWWKEVGLTNKLSFARDRLVESFFWSVGMAFEPQYSRLREELTKVFAFVT
BAS30550.1      TLQNDLKDLSRWWKDVGLTNKLSFARDRLVESFFWSVGMAFEPQYSRLREELTKVFAFVT
AAP40638.1      TLQRDLQDMLGWWNNVGLAKRLSFARDRLMECFFWAVGIAREPALSNCRKGVTKAFSLIL
BAF02831.1      YLQRDLQEMLGWWNNTGLAKRLSFARDRLIECFFWAVGIAHEPSLSICRKAVTKAFALIL
UQP00000.1      -------------------------------CFLWTMGLLFQPQFGYFRRTIAKFFALIT
BAS30549.1      SHQEDVKEISRWWKNTGLGEKLSFARDRLMESFLWSAGVMFQPQYGYSRRIFTKIFALIT
                                                : *: *:  :*  .  *. .:* *.::
BAD98243.1      IIDDIYDVYGTLDELELFTDAVERWDVNAINDLPDYMKLCFLALYNTINEIAYDNLKDKG
AAQ16588.1      IIDDIYDVYGTLDELELFTDAVERWDVNAINDLPDYMKLCFLALYNTINEIAYDNLKDKG
CAL69918.1      IIDDIYDVYGTLDELELFTDAVERWDVNAIDDLPDYMKLCFLALYNTINEIAYDNLKDKG
AEK70970.1      IIDDIYDVYGTLDELELFTDAVERWDVNAINDLPDYMKLCFLALYNTINEIAYDNLKEKG
AAQ84170.1      IIDDVYDVYGTLDELQLFTDAVERWDVNAINTLPDYMKLCFLALYNTVNDTSYSILKEKG
CUR52979.1      IIDDVYDVYGTLDELQLFTDAVERWDVNAINTLPDYMKLCYLALYNTVNDTAYSILKEKG
AEK70968.1      IIDDVYDVYGTLDELELFTNAVERWDVNAVDTLPDYMKLCFFALYNTVNDTAYNLLKEKG
AEK70969.1      IIDDVYDVYGTLDELQLFTDAVERWDVNALDTLPDYMKLCFLALYNTVNDTAYSLLRERG
BAS30551.1      VIDDVYDVYGTVDELELFTDAIERWDVQAVQNLPAYMRICFLALYSTVNDLVYETLKERD
BAS30550.1      VIDDVYDVYGTVDELELFTDAIERWDVQAVQNLPDYMRICFLALYNTVNDLVYETLKERD
AAP40638.1      VLDDVYDVFGTLDELELFTDAVRRWHEDAVENLPGYMKLCFLALYNSVNDMAYETLKETG
BAF02831.1      VLDDVYDVFGTLEELELFTDAVRRWDLNAVEDLPVYMKLCYLALYNSVNEMAYETLKEKG
UQP00000.1      VIDDIYDAYGTLEELECFTNAIERW-----------------------------------
BAS30549.1      ILDDVYDVYGTLDELELFTNAIERWDTNTIDQLPYYMKICFLTLHNSINEIAYDILRERG
                ::**:**.:**::**: **:*:.**
BAD98243.1      ENILPYLTKAWADLCNAFLQEAKWLYNKSTPTFDDYFGNAWKSSSGPLQLVFAYFAVVQN
AAQ16588.1      ENILPYLTKAWADLCNAFLQEAKWLYNKSTPTFDDYFGNAWKSSSGPLQLIFAYFAVVQN
CAL69918.1      ENILPYLTKAWADLCNAFLQEAKWLYNKSTPTFDEYFGNAWKSSSGPLQLVFAYFAVVQN
AEK70970.1      ENILPYLTKAWADLCNAFLQEAKWLYNKSTPTFDDYFGNAWKSSSGPLQLVFAYFAVVQN
AAQ84170.1      HNNLSYLTKSWRELCKAFLQEAKWSNNKIIPAFSKYLENASVSSSGVALLAPSYFSVCQQ
CUR52979.1      HNNISYLTKSWCELCKAFLQEAKWSNNKIIPAFNKYLDNASVSSSGVALLAPSYFLVCQE
AEK70968.1      DNNLPYLAKSWSDLCKAFLQEAKWSNNKIIPSFNKYIENASVSSSGGALLTPCYFSIRQ-
AEK70969.1      DNSLPYLAKSWSELCKAFLQEAKWSNKKTIPEFREYLDNASVSSSGGALLTPCYFSLLTQ
BAS30551.1      EYILPYLTKAWADMCKAFLQEKKWTQNEETPSFEDYLENGWRSSSGGVFLVNAYLLMSQD
BAS30550.1      EYILPYITKAWADMCKAFLQEKKWTQNKETPSFEDYLENGWMSSSGGVFLVNSYLLVSQD
AAP40638.1      ENVTPYLTKVWYDLCKAFLQEAKWSYNKITPGVEEYLNNGWVSSSGQVMLTHAYFLSSPS
BAF02831.1      ENVIPYLAKAWYDLCKAFLQEAKWSNSRIIPGVEEYLNNGWVSSSGSVMLIHAYFLASPS
UQP00000.1      ------------------------------------------------------------
BAS30549.1      VNVIPSLRKVWTDLCRSFLLEATWYHKKHTPTFEEYLQNAWVSVLGPSVLVHVYLSITNP
```

**BAD98243.1**	---IKKE----EIENLQKYHDTISRPSHIFRLCNDLASASAEIARGETANSVSCYMRTK-
**AAQ16588.1**	---IKKE----EIENLQKYHDIISRPSHIFRLCNDLASASAEIARGETANSVSCYMRTK-
**CAL69918.1**	---IKKE----EIDNLQKYHDIISRPSHIFRLCNDLASASAEIARGETANSVSCYMRTK-
**AEK70970.1**	---IKKE----EIENLQKYHDIISRPSHIFRLCNDLASASAEIARGETANSVSCYMRTK-
**AAQ84170.1**	QEDISDH----ALRSLTDFHGLVRSSCVIFRLCNDLATSAAELERGETTNSIISYMHEND
**CUR52979.1**	Q-DISDQ----ALHSLTNFHGLVRSSCTIFRLCNDLATSSAELERGETTNSITSYMHEN-
**AEK70968.1**	--DITNQ----ALDSLTNYHGPVRSSCAIFRLCNDLATSAAELERGETTNSITSYMQDN-
**AEK70969.1**	DVAVTSQFHSSTIDSLTNFHGVVRSSCTIFRLCNDLATSAAELERGETTNSITSYMREK-
**BAS30551.1**	---FTKQ----GLESLENYHNLLRWPSIIFRLANDLATSSAELERGETTNSISCIMSDT-
**BAS30550.1**	---ITKQ----GLESLENYHNLLRWPSIIFRLTNDLATSSAELERGETTNSISCIMSDT-
**AAP40638.1**	---LRKE----ELESLEHYHDLLRLPSLIFRLTNDLATSSAELGRGETTNSILCYMREK-
**BAF02831.1**	---IRKE----ELESLEHYHDLLRLPSLIFRLTNDIASSSAELERGETTNSIRCFMQEK-
**UQP00000.1**	--
**BAS30549.1**	---ITEE----TTRFLEEYPNIIRWSSTIFRLANDLETSEDEIERGDVSKSLQCYMHET-

**BAD98243.1**	GISEELATESVMNLIDETWKKMNKEKLGGSL-FAKPFVETAINLARQSHCTYHNGDAHTS
**AAQ16588.1**	GISEELATESVMNLIDETWKKMNKEKLGGSL-FAKPFVETAINLARQSHCTYHNGDAHTS
**CAL69918.1**	GISEELATESVMNLIDETWKKMNKEKLGGSL-FAKPFVETAINLARQSHCTYHNGDAHTS
**AEK70970.1**	GISEELATESVMNLIDETWKKMNKEKLGGSL-FPKPFVETAINLARQSHCTYHNGDAHTS
**AAQ84170.1**	GTSEEQAREELRKLIDAEWKKMNRERVSDSTLLPKAFMEIAVNMARVSHCTYQYGDGLGR
**CUR52979.1**	ETSEEQACKELRNLIDAEWKKMNEERVSNST-LPKAFREIAINMARISHCTYQYGDGLGR
**AEK70968.1**	GISEEQARDELRNLIDAEWKQINRERVFDQT-FPKAFIETAINMARVSHCTYQYGDGLGR
**AEK70969.1**	GVGEEEAREELSKLIDVEWMKLNRERVLDIGPFPKAFMETAVNMARVSHCTYQHGDGLGR
**BAS30551.1**	GLSEESARQHLTNLIEETWKQMNKDTMSGESPFTKPFMETAINLARIAQCQYQHGDGHGN
**BAS30550.1**	GLSEESARQHLRNLIEETWKQMNKGTMSGESPFTKPFMETAINLARIAQCQYQHGDGHGN
**AAP40638.1**	GFSESEARKQVIEQIDTAWRQMNK-YMVDHSTFNRSFMQMTYNLARMAHCVYQDGDAIGA
**BAF02831.1**	GISELEARECVKEEIDTAWKKMNK-YMVDRSTFNQSFVRMTYNLARMAHCVYQDGDAIGS
**UQP00000.1**	--
**BAS30549.1**	GKSQEESRKYISSLIETTWKKMNKERAVGSS-LFQTYVEIGINLARTAQCMYQHGDGLSV

**BAD98243.1**	PDELTRKRVLSVITEPILPFER---
**AAQ16588.1**	PDELTRKRVLSVITEPILPFER---
**CAL69918.1**	PDELTRKRVLSVITEPILPFER---
**AEK70970.1**	PDELTRKRVLSVITEPILPFER---
**AAQ84170.1**	PDYATENRIKLLLIDPFPINQLMYV
**CUR52979.1**	PDYTTENRIKLLLIDPFPIN-----
**AEK70968.1**	PDNTAENRIKLLLIDPFPI------
**AEK70969.1**	PDNTAQNRIKLLLLNPIPS------
**BAS30551.1**	PDTKSKNRVLSLIIDPIK-------
**BAS30550.1**	PDTKSKNRVLSLIIDPIK-------
**AAP40638.1**	PDDQSWNRVHSLIIKPVSLAPC---
**BAF02831.1**	PDDLSWNRVHSLIIKPISPAA----
**UQP00000.1**	-------------------------
**BAS30549.1**	SDRETKDRIQSVLINPIPLR-----

Figure S3. *IspS* DNA sequence and protein sequence alignment. (**a**) DNA sequence of the 165 bp segment of the gene (*IspS*) encoding for the isoprene synthase protein in pin oak (*Quercus palustris*), identified for the first time in this study. (**b**) protein sequence alignment of the pin oak IspS protein segment and known IspS proteins of other species, used for phylogenetic analyses (tree presented in Figure 6). The *Quercus palustris* IspS segment is highlighted in yellow. Amino acid identity in given positions is denoted by the asterisk * at the bottom of the alignment. The amino acid F338 unique to isoprene synthases is highlighted in bold in the novel pin oak segment. Also highlighted is the highly conserved DDXXD sequence.
